# Serum metabolomic profiling identifies taurolithocholic acid as a predictor of HDM-SCIT response in allergic rhinitis: clinical discovery and experimental validation

**DOI:** 10.3389/fimmu.2026.1822573

**Published:** 2026-05-11

**Authors:** Jing Zhang, Zhouxian Pan, Shisu Zhao, Jihan Li, Lin Chen, Yuxuan Jin, Rui Tang, Xiang Gao, Jinlyu Sun

**Affiliations:** 1 Department of Allergy, State Key Laboratory of Complex Severe and Rare Diseases, Peking Union Medical College Hospital, Chinese Academy of Medical Sciences and Peking Union Medical College, Beijing, China; 2 Department of Allergy, The Affiliated Hospital of Qingdao University, Qingdao, Shandong, China

**Keywords:** allergic rhinitis, house dust mite, metabolomics, subcutaneous immunotherapy, taurolithocholic acid

## Abstract

**Background:**

Subcutaneous immunotherapy (SCIT) is a clinically effective and disease-modifying treatment for house dust mite (HDM)-induced allergic rhinitis (AR). Although SCIT provides long-term symptom improvement for most patients, a subset of patients shows inadequate clinical response. The underlying factors contributing to the heterogeneity in treatment efficacy remain unclear, and robust biomarkers capable of predicting SCIT outcomes are still lacking.

**Objective:**

This study aimed to evaluate the clinical efficacy of one-year HDM-SCIT and to identify serum biomarkers associated with therapeutic response, with the goal of improving prediction of SCIT outcomes in patients with AR.

**Methods:**

Eligible patients with HDM-induced allergic rhinitis were prospectively enrolled from two medical centers (Peking Union Medical College Hospital and the Affiliated Hospital of Qingdao University) and subsequently completed one year of HDM-SCIT. Symptom severity was evaluated using the visual analog scale (VAS) and total nasal symptom score (TNSS), and quality of life by the rhinoconjunctivitis quality of life questionnaire (RQLQ) at baseline and one year after treatment. Clinical remission was defined as ≥ 30% improvement of VAS and RQLQ scores. In addition, medication scores and TNSS were also included as secondary endpoints to support the response definition. Paired serum samples collected before and after treatment were analyzed using untargeted metabolomics and targeted bile acid profiling based on liquid chromatography–mass spectrometry. To validate metabolomic findings, an HDM-induced AR mouse model was established, followed by SCIT alone or SCIT combined with taurolithocholic acid (TLCA, a bile acid). Nasal histopathology, T cell subsets in the spleen, and cytokine levels in serum and nasal lavage fluid were assessed.

**Results:**

Of the 32 patients who received one-year HDM-SCIT, 22 were responders and 10 were non-responders. No baseline differences in age, AR duration, comorbidities, total IgE, VAS, TNSS, or RQLQ were found between the two groups. However, responders had significantly greater reductions in VAS, TNSS, and RQLQ post-one-year treatment (p < 0.05). Untargeted metabolomics identified 956 differential metabolites (614 upregulated, 342 downregulated) between the pre-treatment group and post-treatment group, 1,389 (582 up, 807 down) and 1574 (342 up, 1232 down) between responders and non-responders at baseline and post-treatment, respectively. KEGG analysis highlighted bile secretion as a key differential pathway. Bile acid targeted metabolomics revealed TLCA as a potential biomarker associated with HDM-SCIT efficacy. Mouse models confirmed that HDM-SCIT combined with TLCA, particularly at high dose, alleviated nasal mucosal inflammation (reduced epithelial damage, eosinophils), increased splenic CD4^+^Foxp3^+^ regulatory T cells, reduced CD4^+^IL-4^+^ helper T 2 cells and serum IgG1, and dose-dependently decreased serum/nasal lavage interleukin (IL)-5, while increasing serum IL-10/interferon-γ.

**Conclusion:**

Serum TLCA levels were associated with clinical response to HDM-SCIT. Animal model validation demonstrated that TLCA may enhance SCIT-induced immune tolerance. These findings support TLCA may serve as a potential metabolite-based biomarker and adjunct target for improving the efficacy of allergen immunotherapy.

## 1 Introduction

Allergy to house dust mites (HDM) is a perennial respiratory condition that affects more than half a billion people worldwide (1). HDM are the most prevalent indoor allergens in patients with asthma and/or rhinitis in China. Dermatophagoides pteronyssinus and Dermatophagoides farinae, the two major HDM species, are dominant sources of indoor allergens that trigger allergic inflammation. Successful allergen immunotherapy (AIT) induces an immunologic tolerance to allergens and represents a disease-modifying treatment for allergic airway diseases, such as asthma and rhinitis (2, 3). The best strategy for treating allergic diseases is the combinational approaches including avoiding allergens, patient education, appropriate drug therapy, and AIT. AIT is recommended by the Global Strategy for Asthma Management and Prevention as a treatment option if allergy plays a prominent role, e.g., asthma with allergic rhinoconjunctivitis (4). The key mechanisms underlying AIT include the upregulation of suppressive actions of interleukin (IL)-10 and transforming growth factor-β secreted by regulatory cells, including regulatory T cells (Tregs) and regulatory B cells (Bregs), and isotype switching from immunoglobulin (Ig) E toward IgG4.

AIT is a unique form of therapy in which allergens are administered via the subcutaneous or sublingual route to render long-term relief of symptoms (5, 6). In addition to improving symptoms, reducing medication requirement, and improving quality of life, AIT can change the course of allergic disease progression and induce allergen-specific immune tolerance (7). The use of AIT has increased in China, along with growing evidence from real-world clinical practice. House dust mite subcutaneous immunotherapy (HDM-SCIT) is a therapeutic option for allergic rhinitis (AR) patients who are unable to manage symptoms with standard medications (8). Although multiple global clinical trials have demonstrated the efficacy and safety of SCIT in treating HDM-induced AR, a small subset of patients show inadequate response. However, the underlying mechanism of this heterogeneous response remains poorly understood.

Metabolomics, an emerging field, serves as a novel tool for deciphering variations in treatment response. By integrating advanced high-throughput analytical techniques (including mass spectrometry and nuclear magnetic resonance spectroscopy) with bioinformatics, it investigates changes of metabolite profiles. Characterized by high throughput, high sensitivity, and high specificity, this discipline represents a cutting-edge approach in biomedical research. Serum metabolite detection is convenient, minimally invasive. It can reflect the physiological and pathological state of the body, therefore, with great potential in disease diagnosis, efficacy evaluation, and mechanism research. Multiple studies have demonstrated associations between bile acid metabolism and atopic diseases. Kelly et al. found that asthma is associated with elevated taurine levels (p<0.05), a bile salt involved in fat emulsification, while reduced biliverdin levels (p<0.05) were observed (9). Crestani et al. identified that children with food allergy and asthma displayed significant differences in the ratios of secondary bile acids (10). Huang et al. demonstrated significantly reduced glucuronate and three major conjugated bile acids in the sera of children with atopic dermatitis (11). In this study, we conducted untargeted metabolomics and bile acid targeted metabolomics analyses in HDM-SCIT patients to better characterize metabolic changes associated with clinical response. We aimed to identify potential biomarkers that can influence HDM-SCIT efficacy and to provide supporting evidence through an AR mouse AIT model.

## 2 Methods

### 2.1 Clinical study

#### 2.1.1 Study designs and participants

Adult patients with AR who were mono-sensitized to HDM were recruited from Peking Union Medical College Hospital and Affiliated Hospital of Qingdao University between January 2022 and December 2023. This study was approved by the ethics committees of both hospitals. Informed consent was obtained from all participants (Ethics approval No. QYFYWZLL30725 and No. JS-3353).

The diagnosis of AR was based on the Allergic Rhinitis and its Impact on Asthma guideline (12). Atopic status was determined through a skin prick test and specific serum IgE testing using ImmunoCAP (Phadia, Uppsala, Sweden). See the for Details about inclusion and exclusion criteria are included in supporting information.

**Figure 1:**
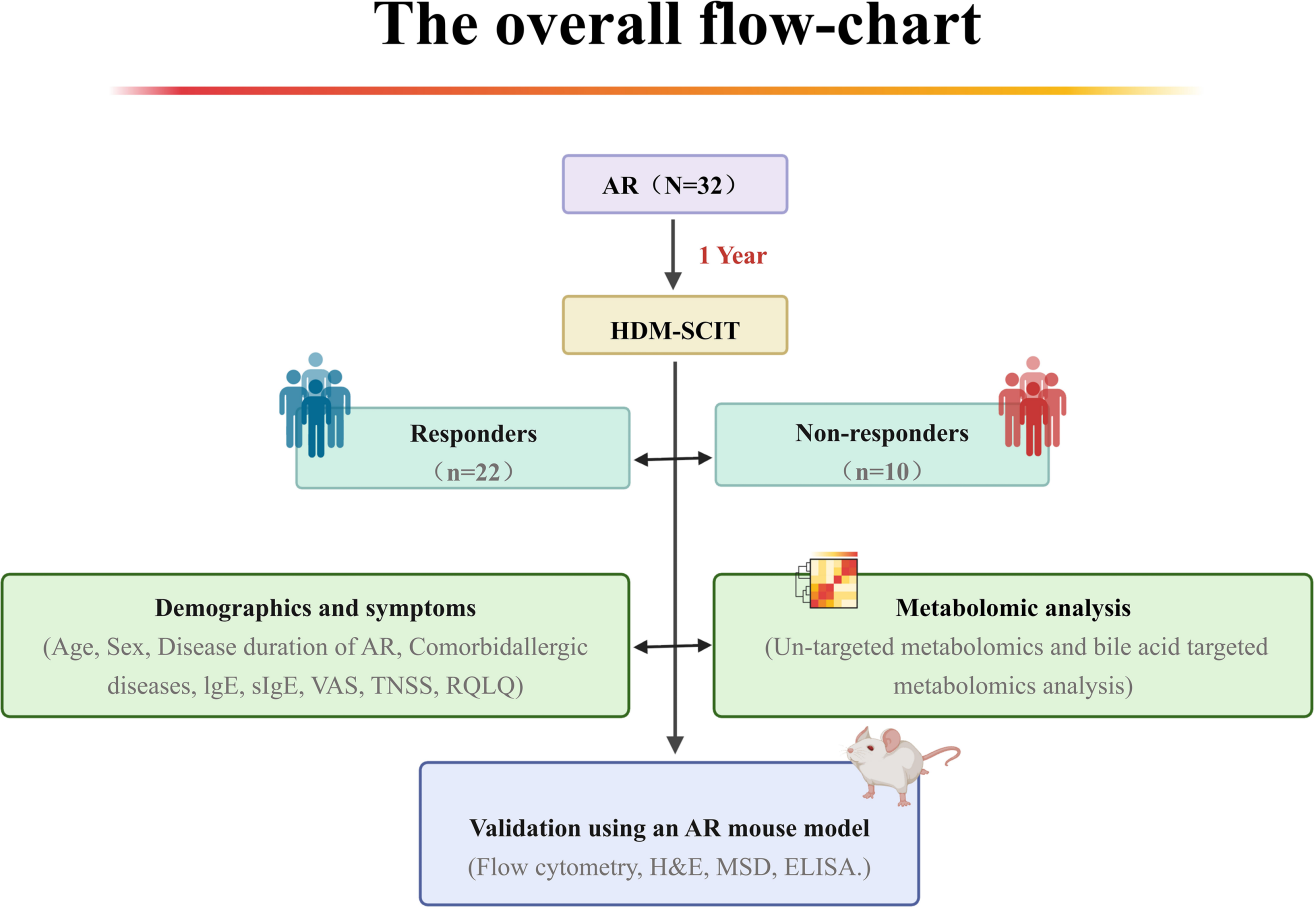
The overall flow-chart. A total of 32 patients received 1 year of HDM-SCIT (Alutard SQ). They were divided into responders and non-responders based on therapeutic efficacy. Demographic information was collected and metabolomics analysis was conducted. The identified potential biomarkers were validated using an AR mouse model with Flow cytometry, HE staining, MSD, and ELISA. AR, allergic rhinitis; HDM, house dust mite; SCIT, subcutaneous immunotherapy; tIgE, total Immunoglobulin E;sIgE, specific Immunoglobulin E; VAS, visual analog scale; TNSS, total nasal symptom score; RQLQ, rhinoconjunctivitis quality of life questionnaire; H&E, hematoxylin and eosin; MSD, Meso Scale Discovery; ELISA, Enzyme linked immunosorbent assay.

The study cohort includes 32 AR patients undergoing SCIT. All eligible patients received HDM-SCIT for one year. HDM-SCIT was administered using the Alutard SQ (ALK-Abello A/S, Hörsholm, Denmark), following the conventional up-dosing scheme (**Supplementary Table 1**), followed by maintenance injections of 100,000 SQ-U every 6 weeks. Conventional pharmacotherapy was continued for all patients throughout the study as recommended by health providers. The overall workflow is shown in **Figure 1**.

#### 2.1.2 Evaluation of SCIT efficacy and definition of responders

Clinical efficacy was assessed using visual analog scale (VAS) (13), total nasal symptom score (TNSS) (14), and rhinoconjunctivitis quality of life questionnaire (RQLQ) before and after one year of HDM-SCIT (15) (**Supplementary Figures 1**-**3**). Clinical response was defined as ≥ 30% improvement in VAS and RQLQ compared to baseline (16–18). In addition, improvement in medication scores and TNSS was also included as a secondary endpoint to support the response definition. This approach was consistent with established clinical trial methodologies for allergen immunotherapy, in which combined symptom and medication scores were recognized as the gold standard for efficacy assessment, while TNSS and medication scores served as validated individual endpoints (19). Patients meeting this criterion were categorized as responders; others were considered as non-responders.

#### 2.1.3 Serum collection

Serum samples from each participant were collected after overnight fasting at the outpatient clinics of the Peking Union Medical College Hospital and the Affiliated Hospital of Qingdao University, both before and one year after HDM-SCIT. The serum samples were immediately frozen at −80 °C until analysis.

#### 2.1.4 Untargeted metabolomic analysis and targeted bile acid metabolomics

##### 2.1.4.1 Serum UHPLC-OE-MS untargeted metabolomics analysis

Untargeted metabolomic profiling was performed using a Vanquish ultra-high-performance liquid chromatography (UHPLC) system (Vanquish, Thermo Fisher Scientific) with a UPLC HSS T3 column (2.1 mm × 100 mm, 1.8 μm) coupled to Orbitrap Exploris 120 mass spectrometer (Orbitrap MS, Thermo). The mobile phase consisted of 5 mmol/L ammonium acetate and 5 mmol/L acetic acid in water (A) and acetonitrile (B). The Orbitrap Exploris 120 mass spectrometer was used for its ability to acquire MS/MS spectra on information-dependent acquisition mode in the control of the acquisition software (Xcalibur, Thermo). (Details in **Supplementary Material**).

##### 2.1.4.2 Bile acid targeted metabolomics analysis

Bile acid targeted metabolomic profiling was performed using an ultra-high-performance liquid chromatography-tandem mass spectrometric (UPLC-MS/MS) (Waters, Milford, MA, USA) equipped with a BEH C18 column (2.1 × 100 mm, 1.8 μm, Waters, Milford, MA, USA). The mobile phases consisted of 0.1% formic acid + water + 10 mM ammonium formate and acetonitrile (phase B). This project utilizes MassLynx 4.1 software for data acquisition and integration. (See **Supplementary Material** for detail).

### 2.2 Mouse model experiments

To validate the clinical metabolomic findings and explore the mechanistic role of taurolithocholic acid (TLCA, a bile acid) in SCIT efficacy, an HDM-induced AR mouse model was established where mice received SCIT with or without TLCA.

#### 2.2.1 Establishment of HDM-induced allergic rhinitis mouse model

Female BALB/c mice (5–6 weeks) were purchased from Beijing Sibeifu Beijing Biotechnology Co. Ltd. (Beijing, China). The Institutional Animal Care and Use Committee of Beijing Shenrui Biotechnology Co, LTD approved all experiments in accordance with their Guide to the Care and Use of Laboratory Animals (Approval No. SR20250415). The mice were housed under specific pathogen-free-conditions. Details of all reagents are provided in **Supplementary Material**.

A total of 25 mice were randomly divided into five groups (n = 5 mice/group): a control group (mice sensitized with saline), a model group (mice sensitized with Dermatophagoides pteronyssinus (GREER, Lenoir, USA), an SCIT group, and two treatment groups receiving SCIT combined with taurolithocholic acid (TLCA, MedChemExpress, USA) administered at doses of 5 mg/kg (low dose) or 10 mg/kg (high dose) (referred to as the SCIT+TLCA5 and SCIT+TLCA 10 groups, respectively). TLCA was administered via intragastric (i.g.) gavage at dose of 5 and 10 mg/kg, based on previous *in vivo* studies in mice (20–22). This dose range was selected due to its established safety profile and bioavailability in rodent models, ensuring appropriate exposure for evaluating its adjunctive effects on SCIT.

**Figure 2:**
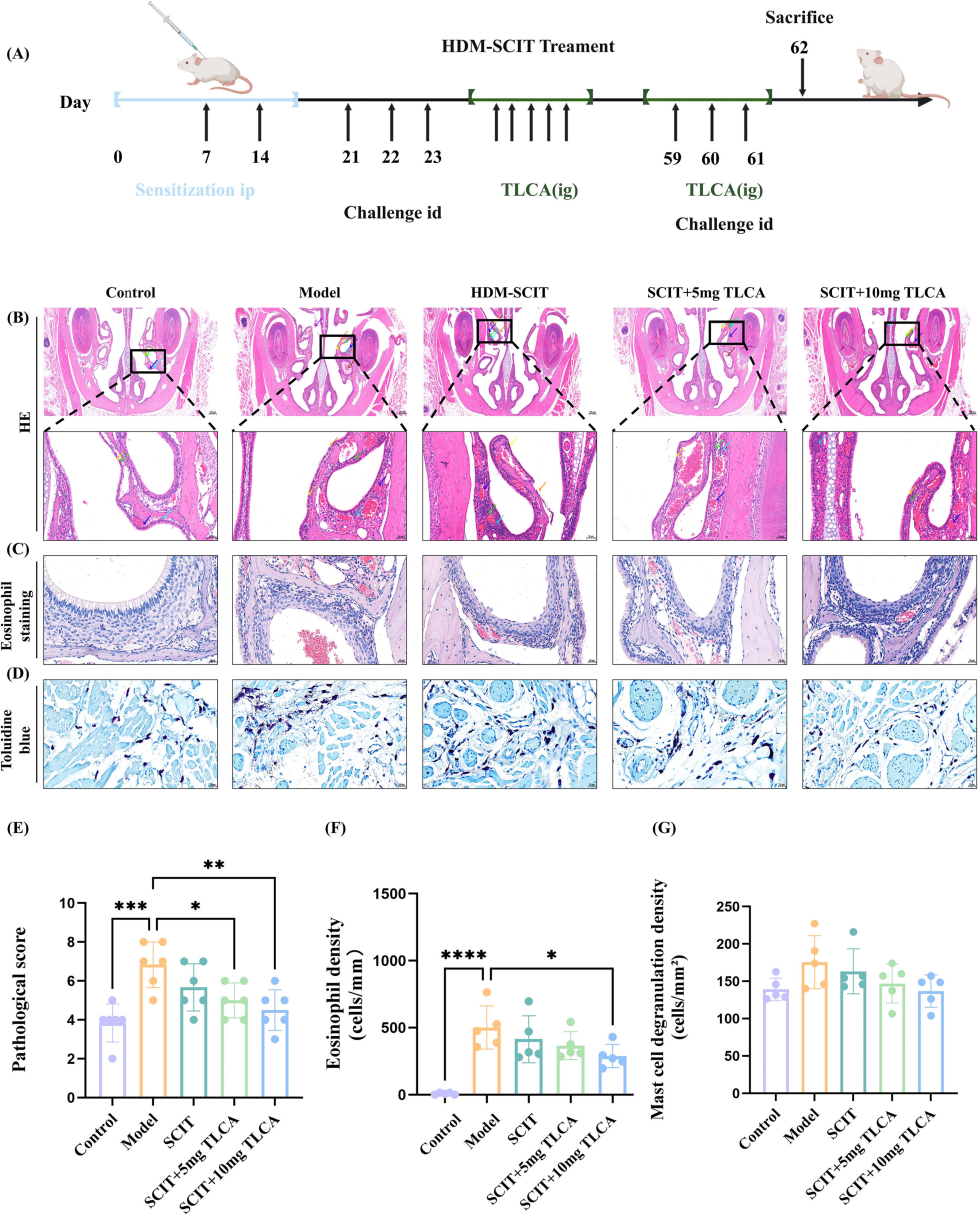
Effects of HDM-SCIT and TLCA on histology of nasal mucosa of dust mite induced allergic rhinitis mouse model. **(A)** Protocol of HDM-SCIT combine with TLCA in a murine model of allergic rhinitis. **(B)** The pathological changes of the nasal mucosa tissue in different groups were observed via H&E staining (4.0×and 20.0×). (the orange arrows indicate epithelial cells shed, the green arrows indicate glands, the cyan arrows indicate granulocyte infiltration, the yellow arrows indicate epithelial cell cilia loss, the blue arrows indicate venous congestion, the purple arrows indicate hemorrhage, the brown arrows indicate acidophilic substance). **(C)** Eosinophil staining analysis of nasal tissues in different groups. **(D)** Analysis of mast cells stained with toluidine blue in nasal tissues of different groups. **(E)** The pathological scoring of H&E staining for different groups was shown in the section. **(F)** The eosinophil density results for different groups were shown in the section, Eosinophil density = eosinophil count/tissue area. **(G)** The degree of mast cell degranulation was shown in the section, the degranulation density of mast cells was calculated by dividing the number of degranulated cells by the field of view area. The histogram was shown as means ± SD (n=5), ****represented this group vs. the model group, p < 0.0001;*** represented this group vs. the model group, p < 0.001;** represented this group vs. the model group, p < 0.01; * represented this group vs. the model group, p < 0.05. ip, intraperitoneal injection; id, intranasal drip; i.g, intragastrical administration; TLCA, Taurolithocholic acid; H&E, hematoxylin and eosin.

BALB/c mice in the model, SCIT, SCIT+TLCA 5 and SCIT+TLCA1–0 groups were sensitized three times by intraperitoneal injection of crude HDM extract (100 μg in 100 μL per mouse per dose) emulsified in aluminum hydroxide gels adjuvant (Biodragon, China) on day 1, 7 and 14. Then, the mice were challenged with intratracheal instillation of HDM extract (25 μg in 20 μL per mouse per dose) extracts on days 21, 22, 23, 59, 60 and 61. The treatment group received SCIT of HDM extracts and i.g. administration TLCA on days 33, 37, 41, 45, 49. Additionally, TLCA was administered during days 59 to 61. All the mice were sacrificed on day 62, and the samples were collected (**Figure 2A**).

#### 2.2.2 Histological analysis of nasal tissue sample

On the day of sacrifice, the entire inferior turbinate of the mouse was excised using dissecting scissors and placed in a general-purpose neutral tissue fixative (Servicebio, Wuhan, China) for histological analysis. After fixation and decalcification, nasal tissues were embedded in paraffin and sectioned at 5 μm slices. The slices were stained with hematoxylin and eosin (H&E) and toluidine blue. H&E staining was used to analyze the morphology and structure of the nasal tissues. Toluidine blue staining was used to observe mast cell degranulation in nasal tissues. Eosinophils were additionally stained and their numbers in the nasal mucosa were quantitated. All staining reagents were purchased from Wuhan Servicebio Technology Co., Ltd (Wuhan, China). Slides were scanned using an LG-S80 digital pathological section scanner (Servicebio, Wuhan, China). Data were analyzed using Saiviewer-1.0.9 software at magnifications ranging from 1× to 400×. Image analysis was performed using Image-Pro Plus 6.0 software. The histological scoring was conducted using a method modified from the International Harmonization of Nomenclature and Diagnostic Criteria for Lesions in Rats and Mice (P. Greaves) (**Supplementary Table 2**).

#### 2.2.3 *In vitro* stimulation of splenic T cells and flow cytometry analysis

The spleen was collected and placed in Cell Staining Buffer (Biolegend, San Diego, CA) on ice until ready for use. Spleens were dissociated into single-cell suspensions using 70 μm cell strainers and cultured in RPMI 1640 supplemented with 10% fetal bovine serum at a concentration of 1.0 × 106 cells/ml.

For flow cytometric analysis of Tregs, a mouse regulatory T-cell staining kit (MultiSciences, Hangzhou, China) was used. After obtaining a single-cell suspension from the spleen, anti-mouse CD4 antibody conjugated with fluorescein isothiocyanate and anti-mouse CD25 antibody conjugated with allophycocyanin were added to the samples. A 1× fixation/permeabilization working solution was then added to the cells. After centrifugation, the cell pellets were resuspended in 1× permeabilization buffer and stained with anti-mouse FOXP3-PE antibody. Splenic CD4^+^ CD25^+^ FOXP3^+^ Tregs of BALB/c mice was determined via flow cytometry.

A mouse helper T cell (Th1/Th2) staining kit (MultiSciences, Hangzhou, China) was used to identify Th1 and Th2 cells in the spleen. PMA/ionomycin and BFA/monensin mixtures were added to the samples. Cells were incubated at 37 °C for 4 hours. After centrifugation and resuspension, anti-mouse APC-Cy7-CD4 antibody was added to the cells. Following treatment with FIX & PERM, anti-mouse FITC-interferon (IFN)-γ and anti-mouse APC-IL-4 antibodies were added to the cells. Splenic Th1 cells (CD4^+^ IFN-γ^+^) and Th2 cells (CD4^+^ IL-4^+^) of BALB/c mice were determined via flow cytometry.

#### 2.2.4 Enzyme-linked immunosorbent assay

Serum levels of IgE (Elabscience Biotechnology, Wuhan, China), IgG1 (MultiSciences, Hangzhou, China), IgG2a (MultiSciences, Hangzhou, China) were measured using the corresponding ELISA kits. The absorbance at wavelengths of 450 and 570 nm was used for measurement.

#### 2.2.5 2.2.5.Cytokine detection in serum and nasal lavage fluid

Approximately 500–600 μL of blood was collected from the ophthalmic artery of each mouse via the orbital artery. The blood samples were stored at 4 °C for 1 hour and then centrifuged at 3000 rpm 15 minutes and 4 °C to obtain serum. For nasal lavage, the nasal cavity of each mouse was flushed twice with 100 μL pre-cooled PBS (BasalMedia, Shanghai, China) using a 1 mL syringe and alveolar lavage needle. A total of 200 μL nasal lavage fluid was collected and centrifuged at 600 × g for 10 minutes; then 100 μL of the supernatant was collected. The levels of inflammatory cytokines in serum and nasal lavage fluid were determined using the Meso Scale Discovery (MSD) electrochemiluminescence assay.

### 2.3 Statistical analysis

All data are expressed as the mean ± SEM, and statistical significance was determined using one-way analysis of variance (ANOVA) or Student’s two-tailed t-test. Categorical data were analyzed using the χ^2^ test. SPSS version 25.0 and GraphPad Prism 10.4.1 software were used for statistical analysis, and FlowJo version 10.8.1 software was employed for flow cytometric fluorescence sorting analysis. Significance levels of data are denoted as * p < 0.05, ** p < 0.01, *** p < 0.001, and **** p < 0.0001.

## 3 Results

### 3.1 Clinical study

#### 3.1.1 Clinical characteristics and treatment efficacy evaluation

Demographic and clinical characteristics of all participants are summarized in **Table 1**. Based on changes in the VAS score, patients were classified into responders (n = 22) and non-responders (n = 10). No statistical differences were observed between the two groups at baseline in age, duration of AR, comorbid allergic diseases, total IgE levels, VAS, TNSS, and RQLQ scores. However, after one year of HDM-SCIT, responders exhibited significantly greater reductions in VAS, TNSS, and RQLQ scores than non-responders (p < 0.05). Further descriptive analysis revealed that non-responders had a higher proportion of comorbid atopic dermatitis (50.0%, 5/10) compared with responders (18.18%, 4/22). Non-responders also showed higher rates of co-sensitization to grass pollen (40.0% vs. 4.55%) and tree pollen (30.0% vs. 9.09%). These observations suggest that comorbid atopic dermatitis and pollen co-sensitization may be potential confounding factors influencing SCIT response.

**Table 1:** Demographic, clinical, and serologic characterization of responders and non-responders of AR patients with SCIT.

	Total	Responders	Non-responders	p Value
Subject number (n)	32	22	10	-
Age (years)	41.22 ± 13.49	43.64 ± 14.49	38.90 ± 9.55	0.085
Gender (male), n (%)	18(56.25)	12(54.55)	6(60.00)	0.79
Disease duration of Allergic rhinitis (years)	5.27 ± 6.00	5.25 ± 6.24	5.30 ± 5.74	0.938
Comorbid allergic diseases, n (%)				
Asthma	11(34.38)	8(36.36)	3(30.00)	-
Atopic dermatitis	9(28.13)	4(18.18)	5(50.00)	-
Chronic urticaria	3(9.38)	2(9.09)	1(10.00)	-
Serum specificity IgE (kU/l), median (range)				
Dermatophagoides pteronyssinus	24.87 ± 29.91	23.56 ± 30.35	27.74 ± 30.31	0.721
Dermatophagoides farina	39.89 ± 33.21	39.66 ± 36.41	40.41 ± 26.56	0.954
Serum totle IgE (kU/l), median (range)	429.75 ± 459.89	369.90 ± 404.94	561.41 ± 563.81	0.282
Co-sensitization pattern, n (%)				
Grass Pollen	5 (15.63)	1 (4.55)	4 (40.00)	-
Animal dander	2 (6.26)	1 (4.55)	1 (10.00)	-
Tree Pollen	5 (15.63)	2 (9.09)	3 (30.00)	-
Fungus	1 (3.13)	1 (4.55)	0 (0.00)	-
Total VAS before HDM-SCIT	7.94 ± 1.13	7.73 ± 0.94	8.40 ± 1.43	0.197
Total VAS after HDM-SCIT	3.56 ± 2.12	2.36 ± 0.71	6.20 ± 1.75	0.000*
Total RQLQ before HDM-SCIT	101.47 ± 27.18	94.05 ± 22.17	114.80 ± 28.42	0.060
Total RQLQ after HDM-SCIT	56.18 ± 32.26	32.50 ± 15.64	92.70 ± 29.82	0.000*
Total TNSS before HDM-SCIT	13.31 ± 2.16	12.91 ± 2.34	14.20 ± 1.40	0.119
Total TNSS after HDM-SCIT	5.84 ± 3.30	4.05 ± 1.43	9.80 ± 2.74	0.000*

IgE, immunoglobulin E; VAS, visual analog scale; TNSS, total nasal symptom score; RQLQ, rhinoconjunctivitis quality of life questionnaire. Significance levels of data are denoted as * p < 0.05.

#### 3.1.2 Untargeted metabolomic analysis characterized the global metabolic changes and enriched pathways associated with HDM-SCIT

To investigate potential factors affecting HDM-SCIT efficacy, we first conducted untargeted metabolomics analysis on AR patients undergoing HDM-SCIT. A total of 16,156 metabolites were detected in serum when comparing the pre-treatment and post-treatment groups. To evaluate whether patient metabolomes differed based on treatment response, patients were classified as responders or non-responders, and their metabolic profiles were analyzed before and after HDM-SCIT. Pre-treatment analysis identified 16,344 metabolites between responders and non-responders. Additionally, 16,294 metabolites were detected between these groups post-treatment (**Table 2**). Univariate analyses, including t-test and fold-change analysis, were used to detect changes in metabolite levels between responders and non-responders. Multivariate statistical analyses were also employed to identify differential metabolites, using criteria of variable importance in the projection (VIP) scores > 1 and p < 0.05. A total of 956 differential metabolites were detected between the pre-treatment group and post-treatment group, with 614 upregulated and 342 downregulated. In the pre-treatment group, 1,389 differential metabolites were identified between responders and non-responders, including 582 upregulated and 807 downregulated. The post-treatment group (responders vs. non-responders) also showed 1,574 differential metabolites between responders and non-responders, with 342 upregulated and 1,232 downregulated (**Table 2**, **Supplementary Figure 4A**). Volcano plots illustrating these differential metabolites are shown in **Supplementary Figure 4A**; down-regulated metabolites in different groups were clustered on the left side while up-regulated metabolites were clustered on the right side.

**Table 2:** Analysis of samples with upregulated and downregulated in differentially expressed metabolites.

Group	Cpd_all	Cpd_diff	Cpd_diff_up	Cpd_diff_down
Pre-treatment vs post-treatment	16156	956	614	342
Non-responders vs Responders (pre-treatment)	16344	1389	582	807
Non-responders vs Responders (post-treatment)	16294	1574	342	1232

Cpd, Compounds Detected; diff, different.

**Figure 3:**
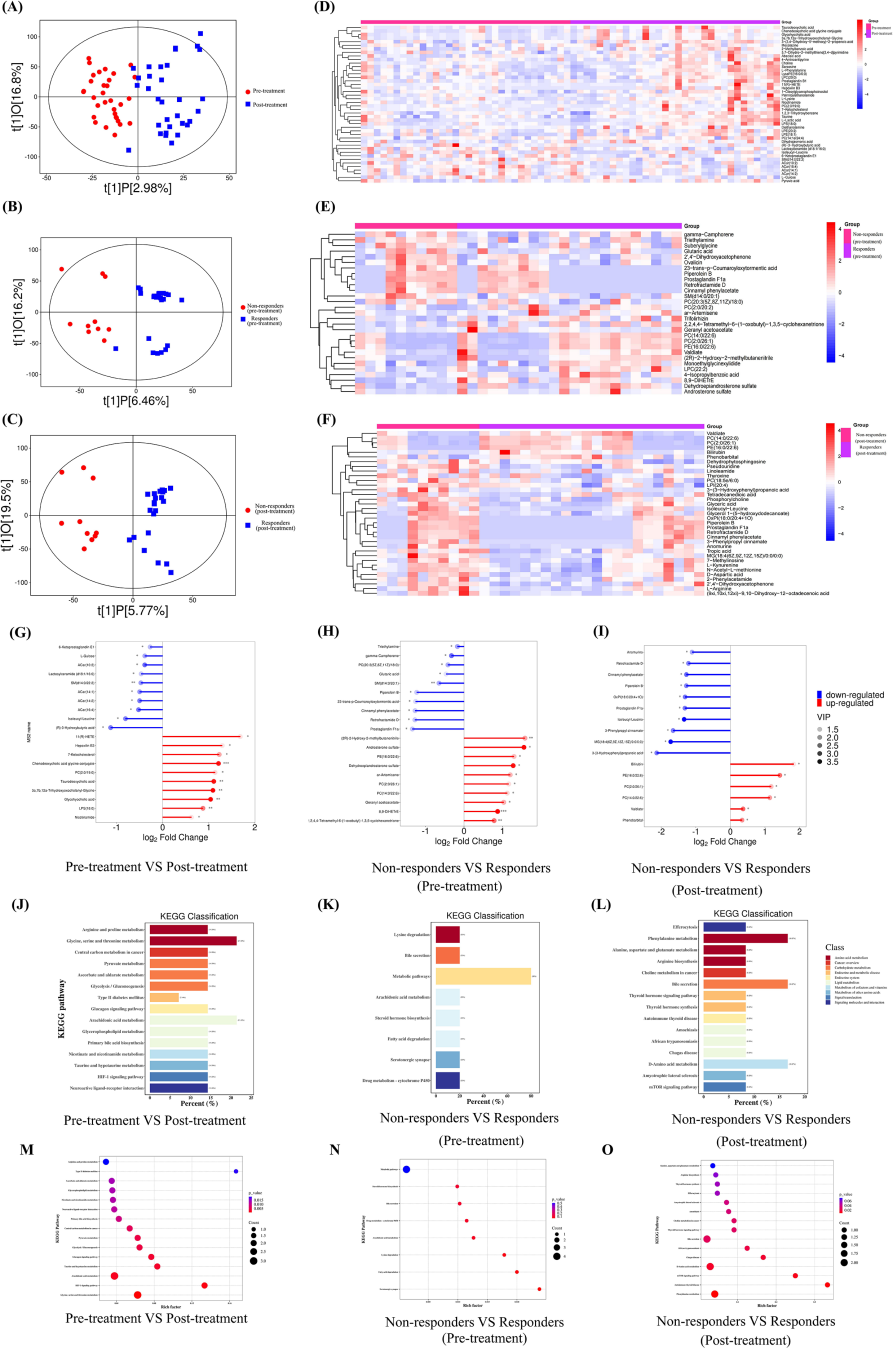
Untargeted metabolomics profiles. **(A–C)** OPLS-DA score plots of the untargeted metabolomics data. **(A)** Pre-treatment (red), post-treatment (blue). **(B)** Non-responders (pre-treatment) (red), Responders (pre-treatment) (blue). **(C)** Non-responders (post-treatment) (red), Responders (post-treatment) (blue). **(D)** Hierarchical clustering analysis of metabolome between pre-treatment (pink) and post-treatment (purple). Red means levels of metabolites increased after HDM-SCIT therapy, while blue is decreased. **(E)** Hierarchical clustering analysis of metabolome between non-responders (pre-treatment) (pink) and Responders (pre-treatment) (purple). **(F)** Hierarchical clustering analysis of metabolome between non-responders (post-treatment) (pink) and responders (post-treatment) (purple). **(G)** The matchstick diagram showed the top 20 significantly changed metabolites in metabolomics analysis based on fold change between pre-treatment and post-treatment. The color depth of the dot represents the VIP value. **(H)** The matchstick diagram showed the top 20 significantly change metabolites in metabolomics analysis based on fold change between non-responders (pre-treatment) and responders (pre-treatment). **(I)** The matchstick diagram showed the top 20 significantly change metabolites in metabolomics analysis based on fold change between non-responders (post-treatment) and responders (post-treatment). **(J)** The KEGG pathway analysis between pre-treatment and post-treatment. **(K)** The KEGG pathway analysis between non-responders (pre-treatment) and responders (pre-treatment). **(L)** The KEGG pathway analysis between non-responders (post-treatment) and responders (post-treatment). **(M)** The KEGG Enrichment bubble analysis between pre-treatment and post-treatment. **(N)** The KEGG Enrichment bubble analysis non-responders (pre-treatment) and responders (pre-treatment). **(O)** The KEGG Enrichment bubble analysis between non-responders (post-treatment) and responders (post-treatment). OPLS-DA, orthogonal partial least squares discriminant analysis; HDM-SCIT, house dust mite subcutaneous immunotherapy; KEGG, Kyoto Encyclopedia of Genes and Genomes. * represents p < 0.05, ** represents p < 0.01, *** represents p < 0.001.

Principal component analysis (PCA), a multivariate statistical technique, was employed to assess whether samples from distinct groups could be differentiated based on their metabolic profiles. Of note, the PCA results did not reveal a clear separation between the groups within the metabolomics dataset (**Supplementary Figure 4B**). Orthogonal partial least squares discrimination analysis (OPLS-DA) was subsequently applied to enhance group discrimination. The OPLS-DA score plots suggested some degree of separation between groups (**Figures 3A–C**). The OPLS-DA model parameters were as follows: for the pre- vs. post-treatment comparison, R²Y(cum) = 0.66 and Q²(cum) = -0.61; for the pre-treatment comparison (responders vs. non-responders), R²Y(cum) = 0.93 and Q²(cum) = -0.28; and for the post-treatment comparison (responders vs. non-responders), R²Y(cum) = 0.77 and Q²(cum) = -0.18 (**Supplementary Figure 4C**). Given the negative Q² values obtained for the comparisons between responders and non-responders, the models inadequately captured inter-group metabolic differences, likely due to the relatively small sample size and the high dimensionality of the metabolomics data. Therefore, these results should be interpreted as exploratory rather than confirmatory. Hierarchical clustering heatmaps visually demonstrated the overall distribution of metabolic differences across groups (**Figures 3D–F**). Additionally, a matchstick diagram illustrated the fold changes in the expression of differential metabolites between the groups (**Figures 3G–I**). These findings indicated that AR patients undergoing HDM-SCIT exhibit significant alterations in their metabolomic biomarker profiles. Notably, these changes occur in both pre- and post-treatment phases across patient populations with varying treatment efficacy. KEGG enrichment analysis results indicate that bile secretion pathways show significant differences across all groups, whether comparing pre- and post-treatment or distinguishing between responders and non-responders (**Figures 3J–O**).

#### 3.1.3 The bile acid targeted metabolomics further explored potential biomarkers associated with HDM-SCIT response

**Figure 4:**
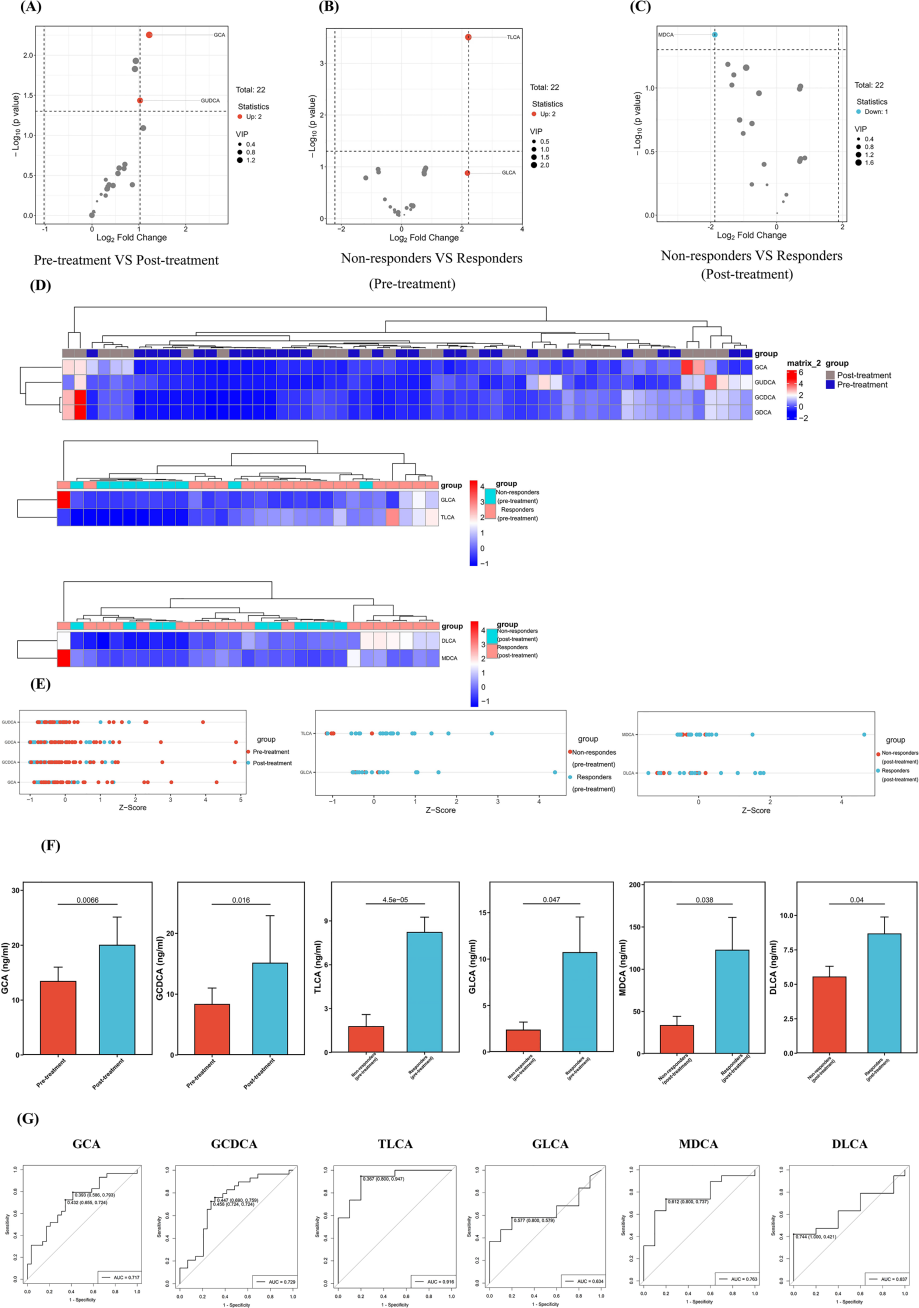
Bile acid targeted metabolomics profiles. **(A–C)** The volcano plot displayed in different groups. **(D)** Heatmaps in different groups. **(E)** Z-scores in different groups **(F)** The columns displayed the differences in bile acid levels across groups (*p* < 0.05). **(G)** ROC curve analysis of bile acid metabolites. ROC, Receiver Operating Characteristic; GCA, Glycocholic acid; GCDCA, Glycochenodeoxycholic acid; GLCA, Glycolithocholic acid; MDCA, Murideoxycholic acid; TLCA, Taurolithocholic acid; DLCA, Deoxycholic acid.

To investigate potential factors affecting HDM-SCIT efficacy, we conducted a bile acid-targeted metabolomics study on these patients based on untargeted metabolic data. The study analyzed 22 types of bile acids, with TLCA showing clear distinctions in between responders and non-responders at both pre- and post-treatment time points (detailed information on 22 bile acids is provided in **Supplementary Table 3**). The volcano plot illustrated up-regulation and down-regulation of bile acids across different groups (**Figures 4A–C**). We constructed hierarchical clustering heatmaps and Z-Score to visually demonstrate the overall distribution of bile acid metabolic differences across different groups (**Figures 4D, E**). **Figure 4F** displays the profiles of bile acids with statistically significant differences between groups (p < 0.05). The levels of GCA, GCDCA were higher in the pre-treatment group. In addition, compared with non-responders, the level of MDCA and DLCA in responders was significantly decreased. Interestingly, at baseline, TLCA levels showed no statistically significant difference between responders and non-responders. However, after one year of HDM-SCIT, TLCA levels were significantly lower in responders than in non-responders (p < 0.001). **Figure 4G** presented the receiver operating characteristic curves for six bile acid metabolites. Among the six bile acid metabolites evaluated, TLCA exhibited the highest diagnostic accuracy with an Area Under Curve (AUC) of 0.916, demonstrating excellent discriminative power for predicting clinical response. GCA, GCDCA, and MDCA showed moderate predictive value (AUC: 0.717–0.763), while GLCA and DLCA had lower AUC values (0.634–0.637), indicating weaker performance. To mitigate the risk of overfitting associated with the limited sample size and to further evaluate the predictive stability of TLCA, we performed multiple internal validation approaches. Bootstrap resampling (1000 iterations) of the TLCA-only model yielded a bias-corrected AUC of 0.9158 (95% CI: 0.7895–1.000), which was nearly identical to the original ROC analysis (AUC = 0.916) (**Supplementary Figure 4D**). This consistency indicates that the predictive performance of TLCA is stable within the current dataset and that the risk of overfitting is low. To further assess the predictive performance of TLCA in a multivariable context, we constructed Random Forest models using two cross-validation strategies: 10-fold cross-validation (10-fold CV) and leave-one-out cross-validation (LOOCV). The 10-fold CV yielded an AUC of 0.7684 (95% CI: 0.5474–0.9579) (**Supplementary Figure 4E**), and LOOCV yielded an AUC of 0.7526 (95% CI: 0.5209–0.9474) (**Supplementary Figure 4F**). Both methods demonstrated moderate predictive ability for TLCA, with the lower bounds of the confidence intervals exceeding 0.5, indicating performance better than random chance. In brief, bile acid targeted metabolomic results indicated that TLCA might serve as a potential biomarker to differentiate the efficacy of HDM-SCIT.

### 3.2 Mouse model experiments

#### 3.2.1 The combination of HDM-SCIT and TLCA effectively alleviates HDM-induced AR-associated nasal mucosal inflammation

Through metabolomic analysis of clinical patients, significant differences in TLCA levels were observed among HDM-SCIT patients with differential treatment efficacy. To investigate the role of TLCA in immunotherapy, we developed a mouse model of AR with and without TLCA treatment. To compare the therapeutic effects of HDM-SCIT alone versus HDM-SCIT combined with TLCA in AR mice, we established a mouse model through HDM sensitization and challenge, followed by HDM-SCIT (**Figure 2A**). Nasal mucosa samples were collected after completion of the modeling procedure, and pathological morphological changes in the nasal mucosa were observed and compared using H&E staining (**Figure 2B**). The results showed that compared with the control group, the experimental group showed more epithelial cell shedding, loss of epithelial cell cilia, increased glandular hyperplasia, and infiltration of lymphocytes and granulocytes in the nasal mucosa. In addition, focal vascular congestion, red blood cell extravasation and eosinophilic material were observed in some mice. The HDM-SCIT group showed partial improvement in nasal mucosal lesions, with further alleviation achieved through combined TLCA therapy. Notably, TLCA administration showed dose dependent enhanced therapeutic effect. **Figure 2E** presents the pathological scores of different groups, with detailed scoring criteria provided in the supporting documents (**Supplementary Table 2**). Additionally, the number of eosinophils in the nasal mucosa and the degree of mast cell degranulation were measured. HDM-SCIT+TLCA group showed significantly lower eosinophil levels (p < 0.05) compared to the model group (**Figure 2C**). However, the SCIT group demonstrated a reduction in eosinophil levels compared to the model group, though this difference was not statistically significant (p > 0.05) (**Figure 2F**). Differences in mast cell degranulation density were also observed among different groups, although not statistically significant compared with the model group (**Figures 2D, G**). In conclusion, these results showed that AR mice exhibited severe nasal mucosa inflammation. HDM immunotherapy could reduce the extent of nasal mucosa damage in AR mice and decrease gland proliferation and infiltration of inflammatory cells. When combined with TLCA, an enhanced efficacy and further reduction of eosinophil levels were observed.

#### 3.2.2 TLCA supplementation altered the population of Th1, Th2 and Treg cells in the spleens of AR mice during HDM-SCIT

**Figure 5:**
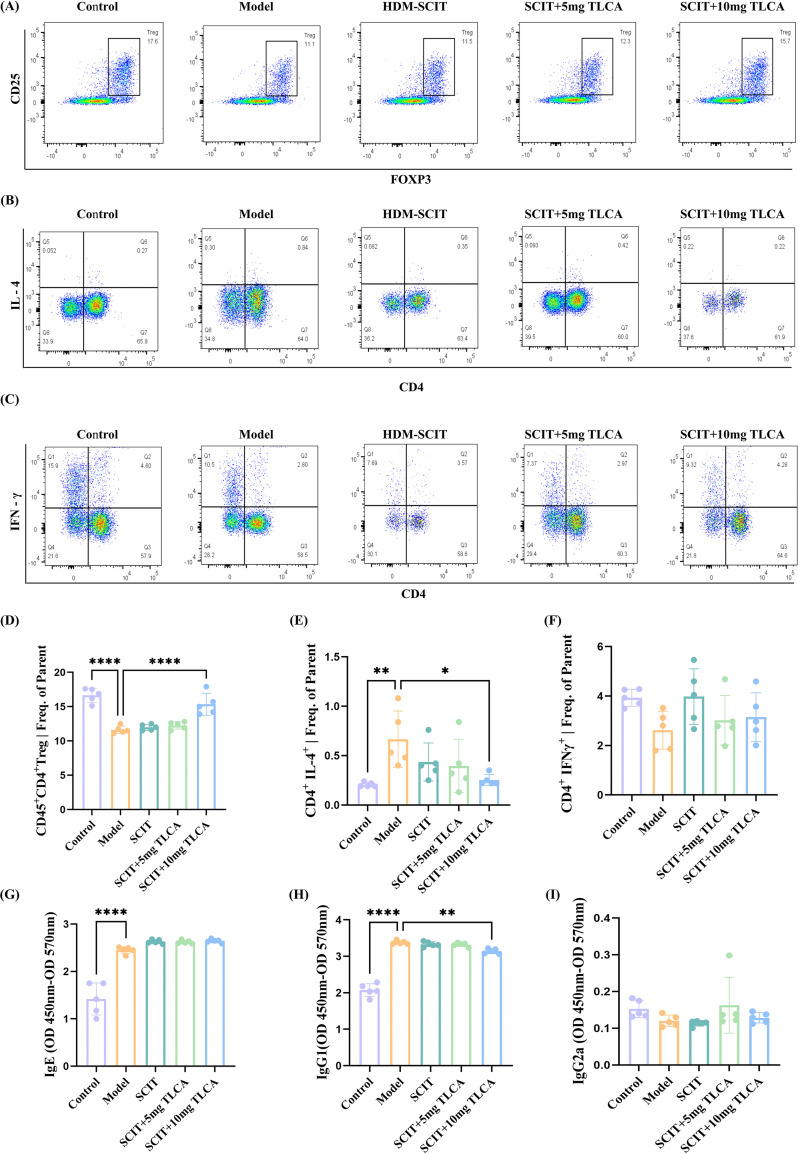
Supplementing TLCA therapy affected the number of Th1, Th2 and Treg cells population from the spleens in AR mice. Flow cytometry was used to determine the number of Th1, Th2 and Treg cells in the spleens of mice in each group. **(A)** The number of Treg cells in the spleens of mice in each group. **(B)** The number of Th2 cells (The circle-gate in Q6 region) in the spleens of mice in each group. **(C)** The number of Th1 cells (The circle-gate in Q2 region) in the spleens of mice in each group. **(D)** The comparison of Lymphocytes/Single Cells/Single Cells/LIVE/CD45^+^/CD4^+^/Treg| Frequency of Parent in different groups. **(E)** The comparison of Lymphocytes/Single Cells/Single Cells/LIVE/CD45^+^/CD3^+^/Q6: CD4^+^, IL-4^+^ | Frequency of Parent in different groups. **(F)** The comparison of Lymphocytes/Single Cells/Single Cells/LIVE/CD45^+^/CD3^+^/Q2: CD4^+^, IFNγ^+^ | Frequency of Parent in different groups. **(G)** ELISA measurement of the difference in absorbance of serum IgE at 450nm and 570nm. **(H)** ELISA measurement of the difference in absorbance of serum IgG1 at 450nm and 570nm. **(I)** ELISA measurement of the difference in absorbance of serum IgG2a at 450nm and 570nm. The histogram was shown as means ± SD (n=5), ****represented this group vs. the model group, p < 0.0001;** represented this group vs. the model group, p < 0.01; * represented this group vs. the model group, p < 0.05.

Flow cytometry was employed to determine the proportions of Th1, Th2 and Treg cells in the spleens of mice across each group (**Figure 5**). Compared with the model group, the percentage of CD4^+^Foxp3^+^Treg cells increased significantly in the SCIT+TLCA10 group (**Figure 5A**). Although Treg levels showed a slight increase after SCIT, the trend became more pronounced following high-dose TLCA supplementation. This phenomenon was not evident in the low-dose TLCA group. CD4^+^ Th1 and Th2 cell subsets were analyzed by the staining of intracellular cytokine IFN-γ and IL-4 CD3^+^CD4^+^. Following high-dose TLCA supplementation, the SCIT+TLCA10 group demonstrated significantly lower levels of CD4^+^IL-4^+^ Th2 cells compared to the model group (**Figure 5B**). However, the level of CD4^+^IFN-γ^+^ Th1 cells showed no significant differences between the model group and other treatment groups, possibly due to insufficient duration of SCIT (**Figure 5C**). These results suggest that TLCA supplementing may enhance the efficacy of SCIT in AR mice by modulating Treg and Th2 cell levels (**Figures 5D–F**).

#### 3.2.3 Effect of TLCA supplementation with HDM-SCIT on IgE, IgG1, IgG2a in the serum of AR mice

In mice, Th2-associated cytokines such as IL-4 promote class switching to IgG1 and IgE, whereas Th1-associated IFN-γ is associated with IgG2a production. Therefore, serum IgE, IgG1, and IgG2a levels were measured by ELISA. Serum IgE levels were significantly higher in the AR group than in the control group (p < 0.0001) (**Figure 5G**). However, no difference was observed between the treatment groups and the model group. Interestingly, serum IgG1 levels were significantly lower in the SCIT+TLCA10 group compared with the model group (p < 0.05) (**Figure 5H**). No statistically significant differences were observed in serum IgG2a levels among the groups (**Figure 5I**).

#### 3.2.4 Effect of TLCA supplementation with HDM-SCIT on cytokines in the serum and nasal lavage fluid of AR mice

**Figure 6:**
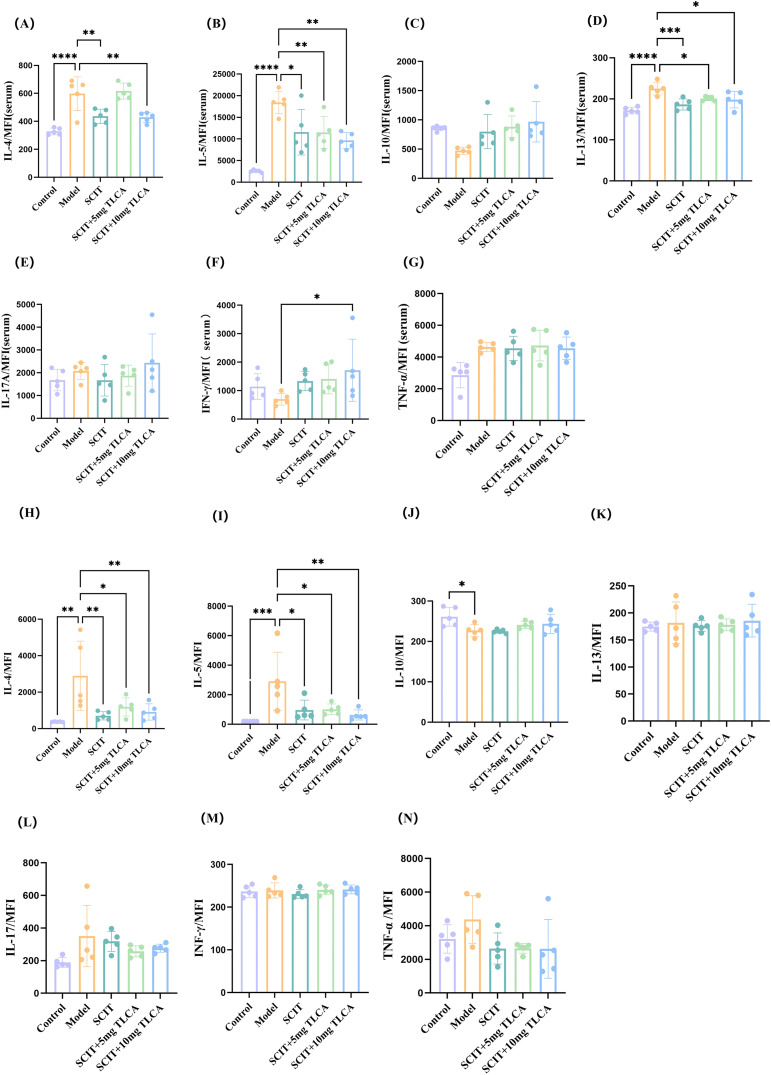
The levels of IL- 4, IL-5, IL-10, IL-13, IL-17A, TNF-α, and IFN-γ in serum and nasal lavage fluid. **(A–G)** The levels of IL- 4, IL- 5, IL-10, IL-13, IL-17A, TNF-α, and IFN-γ in serum, respectively. **(H–N)** The levels of IL - 4, IL- 5, IL- 10, IL-13, IL-17A, TNF -α, and IFN -γ in nasal lavage fluid, respectively. In all plots, solid and dashed bars indicate the median and quartiles, respectively. Each data point represents the MSD results of a single donor. Differences between the groups were analyzed using the Mann - Whitney U test. (n=5) ****represented this group vs. the model group, p < 0.0001;** represented this group vs. the model group, p < 0.01; * represented this group vs. the model group, p < 0.05.

Previous pathological, flow cytometry and ELISA analyses indicated that TLCA supplementation affects Th, Treg, eosinophil levels and IgG1. To further investigate cytokine levels in mouse serum and nasal lavage fluid, MSD technology was employed to detect IL-4, IL-5, IL-10, IL-13, IL-17A, tumor necrosis factor (TNF)-α, and IFN-γ (**Figure 6**). The serum levels of IL-4, IL-5, and IL-13 were significantly lower in the SCIT group and SCIT+TLCA10 group compared to the model group (**Figures 6A, B, D**). Notably, when TLCA was supplemented with SCIT, IL-5 levels decreased further, with dose dependent pattern. However, TLCA supplementation showed no significant improvement in serum IL-4 and IL-13 levels. Furthermore, compared with the model group, IL-10 and IFN-γ levels significantly increased after TLCA supplementation, following a dose-dependent pattern (**Figures 6C, F**). No significant differences were observed in IL-17A and TNF-α levels between treatment groups (**Figures 6E, G**).

Similar to serum results, IL-4 levels in nasal lavage fluid decreased across all three treatment groups compared to the model group. However, TLCA supplementation showed less efficacy than SCIT, likely due to insufficient TLCA dose (**Figure 6H**). IL-5 levels in nasal lavage fluid followed the same trend as serum, with significant reduction after TLCA supplementation (**Figure 6I**). The minimal changes in IL-10, IL-13, IL-17A, TNF-α, and IFN-γ in nasal lavage fluid may reflect insufficient SCIT duration(p < 0.05) (**Figures 6J–N**). Overall, these results demonstrated that co-administration of TLCA with SCIT treatment significantly reduced IL-5 levels in both serum and nasal lavage fluid. Conversely, TLCA supplementation induced marked increases in serum IL-10 and IFN-γ levels, although no change in nasal lavage fluid.

## 4 Discussion

In the present study, we integrated clinical metabolomic profiling with experimental animal model validation to investigate the mechanisms underlying heterogeneous responses to HDM-SCIT in AR. Using an untargeted metabolomics approach followed by targeted bile acid analysis, we identified TLCA might as a metabolite that augmented clinical response to HDM-SCIT. Importantly, the clinical observations were further supported by mechanistic validation in a murine model, in which TLCA supplementation enhanced the immunomodulatory effects of SCIT.

Metabolomics is a systems-level approach enabling high-throughput analysis of biological processes. It has been widely applied in early disease screening, diagnosis, and prognosis, as well as in identifying new drug targets and monitoring therapeutic effects (23, 24). This study employed a systematic framework consisting of clinical metabolomics screening, targeted validation, and animal experiments to elucidate the synergistic effect of TLCA in HDM-SCIT for AR. We characterized the global metabolic alterations in patients with AR before and after one year of HDM-SCIT, using LC-MS-based profiling. Patients were further stratified into responders and non-responders to explore efficacy-associated metabolic differences. Consistent with previous reports, this study revealed that AR patients receiving SCIT exhibited significant improvements in nasal symptoms and quality of life. Moreover, HDM-SCIT induced marked metabolic remodeling after one year of treatment. KEGG pathway enrichment further highlighted bile acid metabolism as a consistently altered mechanism.

To further investigate the relationship between bile acid metabolism and HDM-SCIT efficacy, we conducted bile acid targeted metabolomics on these patients. This analysis revealed significant differences in TLCA levels among patients with varying treatment efficacy. Notably, TLCA levels were significantly lower in non-responders compared to responders at baseline, and such difference sustained after one year of treatment. To confirm these clinical metabolomic findings, we employed an HDM-induced AR model in BALB/c mice. Histological analysis revealed significant inflammatory responses in the nasal mucosa of model mice. Both the SCIT and SCIT combined with TLCA significantly reduced pathological damage and eosinophil levels in nasal tissues, while high-dose TLCA further augmented such effects. These findings demonstrated that TLCA can enhance the anti-inflammatory effects of SCIT in HDM-induced AR.

Flow cytometry analysis further revealed that mice treated with SCIT combined with high-dose (10mg/kg) TLCA exhibited significantly higher levels of CD4^+^CD25^+^FOXP3^+^ Tregs compared with the other two groups, consistent with previous studies in gastric cancer, acute graft-versus-host disease, and esophageal cancer (25). However, no significant up-regulation of splenic Treg cells was observed in mice receiving SCIT alone or SCIT combined with low-dose TLCA (5mg/kg). This may be due to the short duration of SCIT in mice or low antigen dose. Low antigen doses are generally associated with tolerance driven by Treg cells, while high doses tend to induce tolerance via lymphocyte anergy. In line with the observations of Treg cells, CD4+IL-4+Th2 cells displayed significant differences solely in the SCIT+TLCA10 group, reflecting the effect of low HDM dose and short exposure period. Previous studies have shown that prolonged high-dose allergen stimulation during AIT enhances epigenetic regulation of Th2-related genes such as GATA-3, IL-5, and IL-4 (26). Flow cytometric analysis revealed no significant changes in Th1 cell proportions among treatment groups, likely because AIT primarily targets Th2-driven inflammation rather than directly inducing Th1 responses. Alternatively, the treatment dose or duration might be insufficient to induce Th1/Th2 rebalance. Research has indicated that Th1-mediated immune responses are a relatively delayed process, potentially taking months after treatment to manifest (27). The selection of mouse strains critically influences immune response patterns in AR models. Studies have demonstrated significant genetic variations in Th1/Th2 balance among different strains. BALB/c mice predominantly exhibit Th2 responses, whereas C57BL/6 mice tend to show Th1 responses (28). In conclusion, TLCA may work in concert with SCIT to enhance immunomodulation by affecting the differentiation and function of CD4^+^ T cell subsets.

In mice, the most important signature cytokine of Th2 cells is IL-4, which plays a crucial role in the immune response by promoting humoral immunity and allergic reactions. IL-4 and IL-5 induce B-cell class-switching to produce IgE and IgG1. IFN-γ, derived from Th1 cells, stimulates the production of IgG2a antibodies (29, 30). Therefore, we assessed serum levels of IgE, IgG1, IgG2a, and related inflammatory cytokines in mice. SCIT combined with 10mg/kg TLCA significantly reduced serum IgG1 levels compared with the model group. Meanwhile, serum cytokine analysis revealed that the SCIT+TLCA group exhibited significantly lower IL-5 levels compared to the SCIT-only group. The observed reduction is dose-dependent, as supported by the concept of dose-dependent immunomodulation. Conversely, the levels of anti-inflammatory cytokines such as IL-10 and IFN-γ were notably elevated in the SCIT+TLCA group. Th2 cells are one of the primary cellular sources of IL-5, while Th1 cells are the main source of IFN-γ. This finding indicates that TLCA may enhance the therapeutic efficacy of SCIT by regulating the Th1/Th2 immune balance. Notably, IgG2a levels showed no significant differences among groups, consistent with previous Th1 flow cytometry results. Serum IFN-γ level is elevated but not statistically significant, possibly due to the short duration of immunotherapy, not sufficient to stimulate IgG2a production. IL-10, a pleiotropic cytokine, plays a crucial role in the immune system as a regulatory mediator, suppressing allergic inflammatory pathways and modulating excessive immune reactions so to inhibit the development of conditions such as asthma or eczema. Additionally, IL-10 facilitates the induction and enhances the function of Tregs as well as an autocrine cytokine of Tregs (31). However, under chronic stimulation, multiple immune cell types, including effector Th1 and Th2 cells, can produce IL-10, which potentiate AIT. Recent studies suggest the interaction between IL-10 and Treg cells as potential therapeutic mechanism of AIT. Treg cells increase IgG4 production that plays a key role in the immunomodulatory treatment. However, recent studies suggest that inducible type 1 Tregs, primary source of IL-10, rather than Foxp3^+^ Treg cells, contribute to the efficacy of AIT (27, 32). Notably, IL-5 levels in nasal lavage fluid showed significant changes only after TLCA supplementation. IL-5 is important in regulating the proliferation, activation, and maturation of eosinophils. Anti-IL-5 monoclonal antibodies, such as mepolizumab, reslizumab, and benralizumab, have been effectively used to treat eosinophilic asthma. Clinical studies demonstrating their ability to reduce asthma exacerbations and improve lung function (33). This was consistent with previous observations of a reduction in eosinophil numbers in nasal tissue in this patient population.

At the metabolic level, abnormal bile acid metabolism has been identified as a critical factor differentiating the therapeutic response of AR patients to HDM-SCIT. Targeted metabolomics analysis revealed that pre-treatment levels of TLCA were significantly lower in non-responders than in responders, and this difference persisted even after one year of HDM-SCIT. These findings suggest that TLCA levels may serve as a potential biomarker for predicting therapeutic response to HDM-SCIT in AR patients. To assess the robustness of the TLCA prediction model and mitigate the risk of overfitting, we performed internal validation analyses, including bootstrap resampling and Random Forest cross-validation. Nevertheless, internal validation cannot replace independent external validation. Therefore, future studies with larger, multi-center prospective cohorts are needed to independently validate the predictive value of TLCA and confirm its clinical applicability.

At cellular level, TLCA exerted a synergistic effect with SCIT primarily through two core pathways in the high-dose TLCA group, where Treg levels were significantly elevated compared to those in the SCIT-only group. Firstly, it promoted the proliferation of CD4^+^CD25^+^FOXP3^+^ Tregs, potentially enhancing immune tolerance. Secondly, TLCA appeared to modulate the Th1/Th2 immune balance by suppressing Th2/IL-5/eosinophil pathway while enhancing the Th1/anti-inflammatory pathway (increasing serum IFN-γ and IL-10 levels). Additionally, TLCA reduced IgG1 production, further supporting its role in inhibiting Th2-mediated allergic inflammation. Previous studies have demonstrated that TLCA is a nanomolar agonist of the G protein-coupled bile acid receptor TGR5 (GPBAR1) (34, 35), which is widely expressed on immune cells including macrophages, dendritic cells, and T cells. Upon binding, TLCA activates the Gαs-adenylyl cyclase-cAMP-PKA-CREB signaling cascade (36), suppressing pro-inflammatory cytokines and promoting anti-inflammatory phenotypes (37). TLCA downregulates LPS-induced IL-6, TNF, and chemokines in human macrophages and inhibits NF-κB phosphorylation via TGR5 (38). Bile acid metabolites also control Th17 and Treg differentiation through TGR5 and FXR signaling (39, 40) Although direct evidence linking TLCA to Treg/Th2 regulation in SCIT is lacking, these findings suggest that TLCA may indirectly modulate T cell differentiation via the inflammatory microenvironment and antigen-presenting cell function, thereby enhancing the anti-inflammatory efficacy of SCIT.

This study has provided insights in optimization of AR immunotherapy. Mechanistically, this study represents the first to establish a direct connection between TLCA and the therapeutic response of HDM-SCIT. By integrating clinical metabolomics with animal experimental data, the study bridges the gap between metabolic alterations and immune regulation, as evidenced by the comprehensive analysis of metabolic markers and their role in immune monitoring, as well as the validation of findings through the integration of metabolomics data. This study, in conjunction with data from previous studies have underscored the significance of bile acids in immune responses, particularly in allergic diseases such as asthma and food allergy immunotherapy (41–44). The findings further establish bile acids as pivotal regulators in the modulation of allergic inflammation.

This study has several limitations that warrant attention in future research. Firstly, some differences between the human and mouse studies regarding allergen preparation and treatment duration should be acknowledged. Human patients received Alutard SQ, a commercially available, standardized house dust mite (HDM) extract with well-defined allergen content. In contrast, the mouse model utilized crude HDM extract for sensitization and immunotherapy. In previous studies, conventional HDM-SCIT mouse models typically involved 3–5 subcutaneous injections administered over approximately 1–2 weeks (45, 46). This simplified regimen was selected to establish a robust and reproducible experimental system suitable for mechanistic exploration. The SCIT protocol used in the present study—five injections over two consecutive weeks—was designed to model the early phase of the immunotherapeutic response. This approach is consistent with previously reported mechanistic studies on HDM immunotherapy, which have demonstrated that multiple subcutaneous injections of HDM extract over one to two weeks can induce detectable immunological changes, including the induction of regulatory T cells and the suppression of Th2 responses (45, 47). In contrast to the mouse model, human SCIT requires one year of treatment to achieve sustained clinical tolerance (48). This temporal discrepancy reflects inherent differences in lifespan and immune kinetics between mice and humans (49). Nevertheless, acute mouse models remain valuable for investigating the mechanisms underlying early immunomodulatory events, although caution should be exercised when extrapolating findings to long-term clinical outcomes. Furthermore, the animal experimental design was constrained by the relatively short duration of SCIT in BALB/c mice and the use of only a single dose of HDM extract. The interplay between cell death, survival, and low-level steady-state proliferation of Treg cells in the spleen, as observed in untreated mice, could mask the significant changes following SCIT or low-dose TLCA (5 mg/kg) treatments. A longer duration in future studies, treatment cycles and multiple HDM doses should more accurately simulate clinical conditions and validate the long-term effects of TLCA. Secondly, limited clinical sample size and follow-up duration. Although the metabolomic data revealed consistent differences in TLCA levels between responders and non-responders, a larger cohort study with extended follow-up is needed to confirm the stability of TLCA as a predictive biomarker and its correlation with long-term therapeutic outcomes. Thirdly, the precise molecular mechanism through which TLCA regulates Treg cell proliferation and the balance between Th1/Th2 cells remains to be fully clarified. Future studies employing TGR5-specific antagonists, TGR5 knockout mice, or *in vitro* T cell-dendritic cell co-culture systems—combined with transcriptomic or proteomic approaches—are warranted to elucidate the downstream signaling pathways and direct molecular targets associated with the observed immune regulation. Fourth, the present study did not include a TLCA-only group, which limits our ability to determine whether TLCA pretreatment alone can reduce the risk of developing allergic rhinitis. Based on the immunomodulatory effects of TLCA observed in this study (Treg induction, Th2 suppression, reduction of IL-5/eosinophils), it is plausible that TLCA alone might partially attenuate allergic inflammation. However, the effect would likely be less robust than that of SCIT combined with TLCA, as SCIT provides allergen-specific immune modulation. Future studies with a TLCA-only pretreatment group are warranted to test this hypothesis. Finally, the present study found that non-responders had higher rates of comorbid atopic dermatitis and pollen co-sensitization compared with responders. This finding is consistent with previous reports that polysensitized patients respond less favorably to mono-allergen immunotherapy. Therefore, in clinical practice, AR patients with comorbid atopic dermatitis or polysensitization may require multi-allergen immunotherapy or more aggressive pharmacotherapy. However, given the small sample size of non-responders (n = 10), these observations warrant validation in larger cohorts.

## 5 Conclusions

In summary, we employed LC-MS to characterize the global metabolic responses to HDM-SCIT in patients with AR. Metabolomic analysis of serum samples before and after treatment revealed that the level of TLCA is significantly correlated with clinical remission in patients with different therapeutic responses. These findings were further validated by an animal model of HDM-induced AR. TLCA supplementation enhanced the therapeutic efficacy of HDM-SCIT for AR by regulating bile acid metabolism and restoring the Th1/Th2 balance. However, due to the small sample size and the lack of a validation cohort in the study design, further research is required to explore the feasibility of TLCA as a potential predictive biomarker for HDM-SCIT response and a promising adjuvant to improve SCIT treatment outcomes. Future research addressing these current limitations, including expanding the sample size and establishing a validation cohort to enhance the statistical power and generalizability of the findings, will be essential to help validate the clinical applicability of TLCA and to further optimize immunotherapy strategies for AR.

## Data Availability

The original contributions presented in the study are included in the article/**Supplementary Material**. Further inquiries can be directed to the corresponding authors.

## References

[1] Batard T Canonica WG Pfaar O Shamji MH O'Hehir RE . Current advances in house dust mite allergen immunotherapy (AIT): Routes of administration, biomarkers and molecular allergen profiling. Mol Immunol. (2023) 155:124–34. doi: 10.1016/j.molimm.2023.02.004. PMID: 36806944

[2] Głobińska A Boonpiyathad T Satitsuksanoa P Kleuskens M van de Veen W Sokolowska . Mechanisms of allergen-specific immunotherapy: Diverse mechanisms of immune tolerance to allergens. Ann Allergy Asthma Immunology: Off Publ Am Coll Allergy Asthma Immunol. (2018) 121:306–12. doi: 10.1016/j.anai.2018.06.026. PMID: 29966703

[3] Berings M Karaaslan C Altunbulakli C Gevaert P Akdis M Bachert C . Advances and highlights in allergen immunotherapy: On the way to sustained clinical and immunologic tolerance. J Allergy Clin Immunol. (2017) 140:1250–67. doi: 10.1016/j.jaci.2017.08.025. PMID: 28941667

[4] Mallen . (*NEW) 2019 GINA report: global strategy for asthma management and prevention (2018). Available online at: https://ginasthma.org/ (Accessed April 22, 2026).

[5] Jacobsen L Niggemann B Dreborg S Ferdousi HA Halken S Høst A . Specific immunotherapy has long-term preventive effect of seasonal and perennial asthma: 10-year follow-up on the PAT study. Allergy. (2007) 62:943–8. doi: 10.1111/j.1398-9995.2007.01451.x. PMID: 17620073

[6] Agache I Lau S Akdis CA Smolinska S Bonini M Cavkaytar O . EAACI Guidelines on Allergen Immunotherapy: House dust mite-driven allergic asthma. Allergy. (2019) 74:855–73. doi: 10.1111/all.13749. PMID: 31095767

[7] Feng M Zeng X Li J . House dust mite subcutaneous immunotherapy in Chinese patients with allergic asthma and rhinitis. J Thorac Dis. (2019) 11:3616–25. doi: 10.21037/jtd.2019.06.35. PMID: 31559069 PMC6753424

[8] Harintajinda S Klangkalya N Kanchongkittiphon W Rerkpattanapipat T Kerddonfak S Manuyakorn W . Allergic rhinitis in remission with house dust mite subcutaneous immunotherapy. Asian Pacific J Allergy Immunol. (2025) 43:189–97. doi: 10.12932/AP-140224-1785. PMID: 39306739

[9] Kelly RS Sordillo JE Lasky-Su J Dahlin A Perng W Rifas-Shiman SL . Plasma metabolite profiles in children with current asthma. Clin Exp Allergy: J Br Soc For Allergy Clin Immunol. (2018) 48:1297–304. doi: 10.1111/cea.13183. PMID: 29808611 PMC6160355

[10] Crestani E Harb H Charbonnier LM Leirer J Motsinger-Reif A Rachid R . Untargeted metabolomic profiling identifies disease-specific signatures in food allergy and asthma. J Allergy Clin Immunol. (2020) 145:897–906. doi: 10.1016/j.jaci.2019.10.014. PMID: 31669435 PMC7062570

[11] Huang Y Chen G Liu X Shao Y Gao P Xin C . Serum metabolomics study and eicosanoid analysis of childhood atopic dermatitis based on liquid chromatography-mass spectrometry. J Proteome Res. (2014) 13:5715–23. doi: 10.1021/pr5007069. PMID: 25316199

[12] Brożek JL Bousquet J Agache I Agarwal A Bachert C Bosnic-Anticevich S . Allergic Rhinitis and its Impact on Asthma (ARIA) guidelines-2016 revision. J Allergy Clin Immunol. (2017) 140:950–8. doi: 10.1016/j.jaci.2017.03.050. PMID: 28602936

[13] Ciprandi G Mora F Cassano M Gallina AM Mora R . Visual analog scale (VAS) and nasal obstruction in persistent allergic rhinitis. Otolaryngology--Head Neck Surgery: Off J Am Acad Otolaryngology-Head Neck Surg. (2009) 141:527–9. doi: 10.1016/j.otohns.2009.06.083. PMID: 19786224

[14] Aksoy C Elsürer Ç Artaç H Bozkurt MK . Evaluation of olfactory function in children with seasonal allergic rhinitis and its correlation with acoustic rhinometry. Int J Pediatr Otorhinolaryngology. (2018) 113:188–91. doi: 10.1016/j.ijporl.2018.07.051. PMID: 30173982

[15] Juniper EF Guyatt GH Griffith LE Ferrie PJ . Interpretation of rhinoconjunctivitis quality of life questionnaire data. J Allergy Clin Immunol. (1996) 98:843–5. doi: 10.1016/s0091-6749(96)70135-5. PMID: 8876562

[16] Malling HJ . Immunotherapy as an effective tool in allergy treatment. Allergy. (1998) 53:461–72. doi: 10.1111/j.1398-9995.1998.tb04082.x. PMID: 9636804

[17] Chinese Society of Allergy (CSA) Chinese Allergic Rhinitis Collaborative Research Group (C2AR2G) . Chinese guideline on allergen immunotherapy for allergic rhinitis: the 2022 update. Allergy Asthma Immunol Res. (2022) 14:604–52. doi: 10.4168/aair.2022.14.6.604. PMID: 36426395 PMC9709690

[18] Kim JY Jang MJ Kim DY Park SW Han DH . Efficacy of subcutaneous and sublingual immunotherapy for house dust mite allergy: A network meta-analysis-based comparison. J Allergy Clin Immunol In Pract. (2021) 9:4450–4458.e6. doi: 10.1016/j.jaip.2021.08.018. PMID: 34464748

[19] Canonica GW Bachert C Hellings P Ryan D Valovirta E Wickman M . Allergen immunotherapy (AIT): a prototype of precision medicine. World Allergy Organ J. (2015) 8:31. doi: 10.1186/s40413-015-0079-7. PMID: 26594303 PMC4640346

[20] Zheng X Zhang Y Zhang L Yang T Zhang F Wang X . Taurolithocholic acid protects against viral haemorrhagic fever via inhibition of ferroptosis. Nat Microbiol. (2024) 9:2583–99. doi: 10.1038/s41564-024-01801-y. PMID: 39294459

[21] Zhang ZY Guo XL Liu JT Gu YJ Ji XW Zhu S . Conjugated bile acids alleviate acute pancreatitis through inhibition of TGR5 and NLRP3 mediated inflammation. J Transl Med. (2024) 22:1124. doi: 10.1186/s12967-024-05922-0. PMID: 39707318 PMC11662532

[22] Eggink HM Tambyrajah LL van den Berg R Mol IM van den Heuvel JK Koehorst M . Chronic infusion of taurolithocholate into the brain increases fat oxidation in mice. J Endocrinol. (2018) 236:85–97. doi: 10.1530/JOE-17-0503. PMID: 29233934

[23] Spertini F . Metabolomics and allergy: Opening Pandora's box. J Allergy Clin Immunol. (2020) 145:782–4. doi: 10.1016/j.jaci.2020.01.012. PMID: 31981625

[24] Turi KN Romick-Rosendale L Ryckman KK Hartert TV . A review of metabolomics approaches and their application in identifying causal pathways of childhood asthma. J Allergy Clin Immunol. (2018) 141:1191–201. doi: 10.1016/j.jaci.2017.04.021. PMID: 28479327 PMC5671382

[25] Qi Y Duan G Wei D Zhao C Ma Y . The bile acid membrane receptor TGR5 in cancer: Friend or foe? Molecules (Basel Switzerland). (2022) 27:5292. doi: 10.3390/molecules27165292. PMID: 36014536 PMC9416356

[26] Bégin P Nadeau KC . Epigenetic regulation of asthma and allergic disease. Allergy Asthma Clin Immunology: Off J Can Soc Allergy Clin Immunol. (2014) 10:27. doi: 10.1186/1710-1492-10-27. PMID: 24932182 PMC4057652

[27] Möbs C Slotosch C Löffler H Jakob T Hertl M Pfützner W . Birch pollen immunotherapy leads to differential induction of regulatory T cells and delayed helper T cell immune deviation. J Immunol (Baltimore Md: 1950). (2010) 184:2194–203. doi: 10.4049/jimmunol.0901379. PMID: 20048125

[28] Lee KI Bae JS Kim EH Kim JH Lyu L Chung YJ . Strain-specific differences in house dust mite (Dermatophagoides farinae)-induced mouse models of allergic rhinitis. Clin Exp Otorhinolaryngology. (2020) 13:396–406. doi: 10.21053/ceo.2019.01837. PMID: 32407614 PMC7669312

[29] Ebrahimpoor S Pakzad SR Ajdary S . IgG1 and IgG2a profile of serum antibodies to Leishmania major amastigote in BALB/c and C57BL/6Mice. Iranian J Allergy Asthma Immunol. (2013) 12:361–7. 23996712

[30] Waśkiel-Burnat A Osińska M Salińska A Blicharz L Goldust M Olszewska M . The role of serum Th1, Th2, and Th17 cytokines in patients with alopecia areata: Clinical implications. Cells. (2021) 10:3397. doi: 10.3390/cells10123397. PMID: 34943905 PMC8699846

[31] Hawrylowicz CM O'Garra A . Potential role of interleukin-10-secreting regulatory T cells in allergy and asthma. Nat Rev Immunol. (2005) 5:271–83. doi: 10.1038/nri1589. PMID: 15775993

[32] Möbs C Ipsen H Mayer L Slotosch C Petersen A Würtzen PA . Birch pollen immunotherapy results in long-term loss of Bet v 1-specific TH2 responses, transient TR1 activation, and synthesis of IgE-blocking antibodies. J Allergy Clin Immunol. (2012) 130:1108–1116.e6. doi: 10.1016/j.jaci.2012.07.056. PMID: 23021882

[33] Li S Wang S Fordjour E Liang Y Wang X Ye Y . Development and characterization of anti-IL-5 monoclonal antibody Fab fragment for blocking IL-5/IL-5Rα binding. Int Immunopharmacol. (2023) 124:111032. doi: 10.1016/j.intimp.2023.111032. PMID: 37832239

[34] Prawitt J Caron S Staels B . Bile acid metabolism and the pathogenesis of type 2 diabetes. Curr Diabetes Rep. (2011) 11:160–6. doi: 10.1007/s11892-011-0187-x. PMID: 21431855 PMC3338411

[35] Cipriani S Mencarelli A Chini MG Distrutti E Renga B Bifulco G . The bile acid receptor GPBAR-1 (TGR5) modulates integrity of intestinal barrier and immune response to experimental colitis. PloS One. (2011) 6:e25637. doi: 10.1371/journal.pone.0025637. PMID: 22046243 PMC3203117

[36] Pols TWH Puchner T Korkmaz HI Vos M Soeters MR de Vries CJM . Lithocholic acid controls adaptive immune responses by inhibition of Th1 activation through the Vitamin D receptor. PloS One. (2017) 12:e0176715. doi: 10.1371/journal.pone.0176715. PMID: 28493883 PMC5426628

[37] Wammers M Schupp AK Bode JG Ehlting C Wolf S Deenen R . Reprogramming of pro-inflammatory human macrophages to an anti-inflammatory phenotype by bile acids. Sci Rep. (2018) 8:255. doi: 10.1038/s41598-017-18305-x. PMID: 29321478 PMC5762890

[38] Wu S Romero-Ramírez L Mey J . Taurolithocholic acid but not tauroursodeoxycholic acid rescues phagocytosis activity of bone marrow-derived macrophages under inflammatory stress. J Cell Physiol. (2022) 237:1455–70. doi: 10.1002/jcp.30619. PMID: 34705285 PMC9297999

[39] Hang S Paik D Yao L Kim E Trinath J Lu J . Bile acid metabolites control TH17 and Treg cell differentiation. Nature. (2019) 576:143–8. doi: 10.1038/s41586-019-1785-z. PMID: 31776512 PMC6949019

[40] Song X Sun X Oh SF Wu M Zhang Y Zheng W . Microbial bile acid metabolites modulate gut RORγ+ regulatory T cell homeostasis. Nature. (2020) 577:410–5. doi: 10.1038/s41586-019-1865-0. PMID: 31875848 PMC7274525

[41] Nakada EM Bhakta NR Korwin-Mihavics BR Kumar A Chamberlain N Bruno SR . Conjugated bile acids attenuate allergen-induced airway inflammation and hyperresponsiveness by inhibiting UPR transducers. JCI Insight. (2019) 4:e98101. doi: 10.1172/jci.insight.98101. PMID: 31045581 PMC6538331

[42] Siddesha JM Nakada EM Mihavics BR Hoffman SM Rattu GK Chamberlain N . Effect of a chemical chaperone, tauroursodeoxycholic acid, on HDM-induced allergic airway disease. Am J Physiol Lung Cell Mol Physiol. (2016) 310:L1243–59. doi: 10.1152/ajplung.00396.2015. PMID: 27154200 PMC4935467

[43] Arifuzzaman M Won TH Li TT Yano H Digumarthi S Heras AF . Inulin fibre promotes microbiota-derived bile acids and type 2 inflammation. Nature. (2022) 611:578–84. doi: 10.1038/s41586-022-05380-y. PMID: 36323778 PMC10576985

[44] Styles JN Wang JK Raman A Lu M Lasky-Su JA Vickery BP . Association of bile acids, amino acids, and glycerophospholipid metabolites with food-allergic outcomes in children on peanut oral immunotherapy. medRxiv: preprint server Health Sci. (2025), 2025.07.01.25330651. doi: 10.1101/2025.07.01.25330651. PMID: 40630602 PMC12236891

[45] Hesse L van Ieperen N Habraken C Petersen AH Korn S Smilda T . Subcutaneous immunotherapy with purified Der p1 and 2 suppresses type 2 immunity in a murine asthma model. Allergy. (2018) 73:862–74. doi: 10.1111/all.13382. PMID: 29318623 PMC5947840

[46] Lee HJ Kim JA Lee Y Lim S Chun YH . Allergic-specific immunotherapy using injectable in situ crosslinked hyaluronic acid hydrogels ameliorates allergic response in murine allergic rhinitis model. Allergy Asthma Immunol Res. (2025) 17:60–76. doi: 10.4168/aair.2025.17.1.60. PMID: 39895603 PMC11791369

[47] Park YM Kim H Kim JA Lee HJ Chun YH . Intravenous immunotherapy with Der p 1-containing nanoparticles alleviates the allergic responses in a murine allergic rhinitis model. Hum Vaccines Immunotherapeutics. (2026) 22:2612810. doi: 10.1080/21645515.2026.2612810. PMID: 41805230 PMC12977256

[48] Olsen OT Larsen KR Jacobsan L Svendsen UG . A 1-year, placebo-controlled, double-blind house-dust-mite immunotherapy study in asthmatic adults. Allergy. (1997) 52:853–9. doi: 10.1111/j.1398-9995.1997.tb02157.x. PMID: 9284985

[49] Sellers RS . Translating mouse models. Toxicol Pathol. (2017) 45:134–45. doi: 10.1177/0192623316675767. PMID: 27815489

