# Supplementary material for: Serum metabolomic profiling identifies taurolithocholic acid as a predictor of HDM-SCIT response in allergic rhinitis: clinical discovery and experimental validation
Source: Front Immunol. 2026 May 11;17:1822573. doi: 10.3389/fimmu.2026.1822573 (PMC13199366; doi:10.3389/fimmu.2026.1822573)
Supplement: Supplementary file 1 [file SupplementaryFile1.docx]

Supplementary Material

# Methods

## Clinical study

### Participants and samples

Inclusion and exclusion criteria：

- Inclusion criteria for the allergic rhinitis (AR) group

1)The diagnosis of AR was confirmed by a hospital clinician in conjunction with clinical manifestations, serum levels of Dermatophagoides pteronyssinus (Der p) and D. farinae (Der f) -specific IgE greater than 0.7 KU/L, and a positive skin prick test (SPT) response (skin wheal index≥2) to Der p and Der f, and was in accordance with the Chinese Guidelines for the Diagnosis and Treatment of Allergic Rhinitis (2022, Revised Edition)

2)Ages 18 to 60 years

3)The patients must have at least 1 year AR history

4)The patients participating in the study need to stop antibiotics and steroid hormones for more than 4 weeks, including intranasal steroid hormones

- Exclusion criteria

1）Combined sinusitis, nasal polyps, or nasal tumors

2) Combined malignant tumors

3) Combined severe cardiac, hepatic, and renal functional disease

4) Coagulation abnormalities

5) Illiteracy, poor self-awareness and cooperation

6) Comorbid mental illness or severe communication disorders

7) The patients had undergone another SIT immunotherapy within the five years prior to this study.

### House dust mite subcutaneous immunotherapy and symptoms evaluation

**Supplementary** Table 1. List of doses in initial and maintenance treatment stages

|  | Time of injection during treatment/week | Vial | Injection volume  (mL) | Dose （SQ-U） |
| --- | --- | --- | --- | --- |
| Initial treatment phase | 1 | 1 (100 SQ-U/mL) | 0.2 | 20 |
|  | 2 |  | 0.4 | 40 |
|  | 3 |  | 0.8 | 80 |
|  | 4 | 2 (1,000 SQ-U/mL) | 0.2 | 200 |
|  | 5 |  | 0.4 | 400 |
|  | 6 |  | 0.8 | 800 |
|  | 7 | 3 (10,000 SQ-U/mL) | 0.2 | 2,000 |
|  | 8 |  | 0.4 | 4,000 |
|  | 9 |  | 0.8 | 8,000 |
|  | 10 | 4 (100,000 SQ-U/mL) | 0.1 | 10,000 |
|  | 11 |  | 0.2 | 20,000 |
|  | 12 |  | 0.4 | 40,000 |
|  | 13 |  | 0.6 | 60,000 |
|  | 14 |  | 0.8 | 80,000 |
|  | 15 |  | 1.0 | 100,000 |
| Maintenance therapy phase | 17 |  | 1.0 | 100,000 |
|  | 21 |  | 1.0 | 100,000 |
|  | 27 |  | 1.0 | 100,000 |
|  | 33 |  | 1.0 | 100,000 |
|  | 39 |  | 1.0 | 100,000 |
|  | 45 |  | 1.0 | 100,000 |
|  | 51 |  | 1.0 | 100,000 |

### Evaluation of SCIT efficacy and definition of responders

Observation indicators:

The severity of clinical symptoms in AR patients before and after H2 treatment was scored using the VAS,TNSS, and RQLQ.


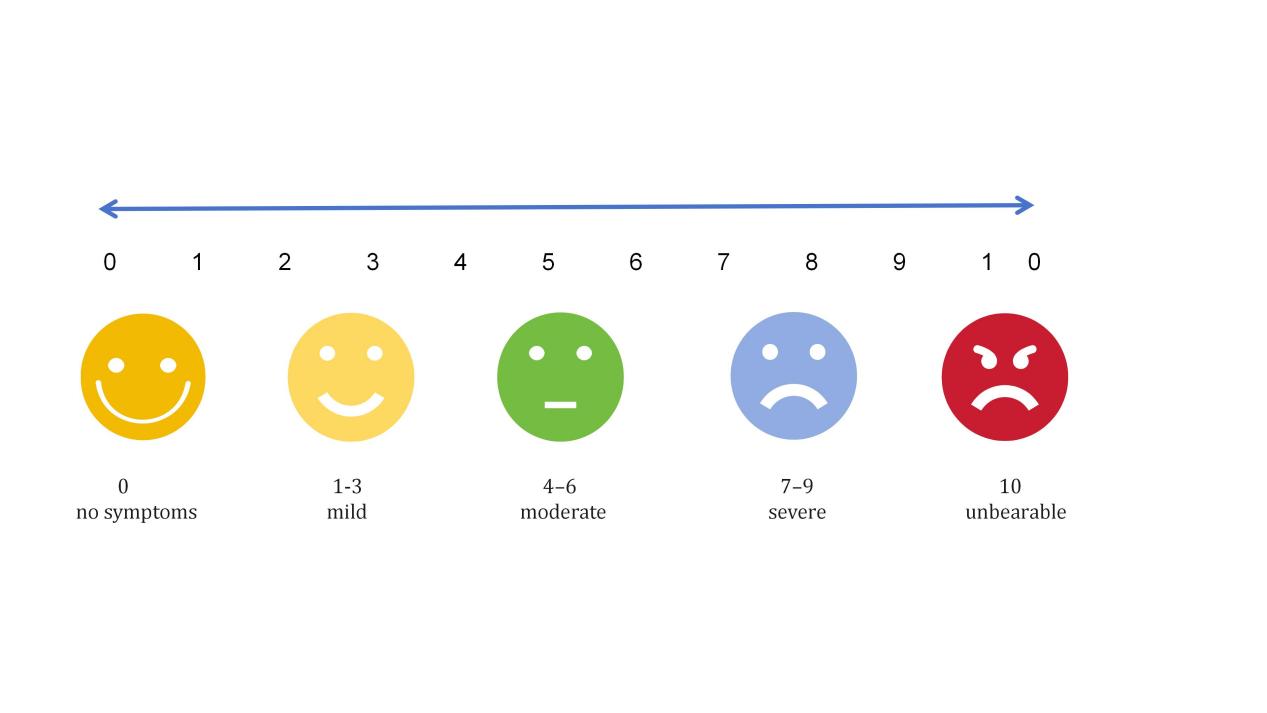


**Supplementary Figure 1.** VAS score: Patients were scored according to their symptoms (nasal congestion, runny nose, itchy nose, sneezing) in the last week using a 0–10 cm analog scale: 0 cm: 0 points, no symptoms; 1–3 cm: 1–3 points, mild; 4–6 cm: 4–6 points, moderate; 7–10 cm: 7–10 points, severe.

**
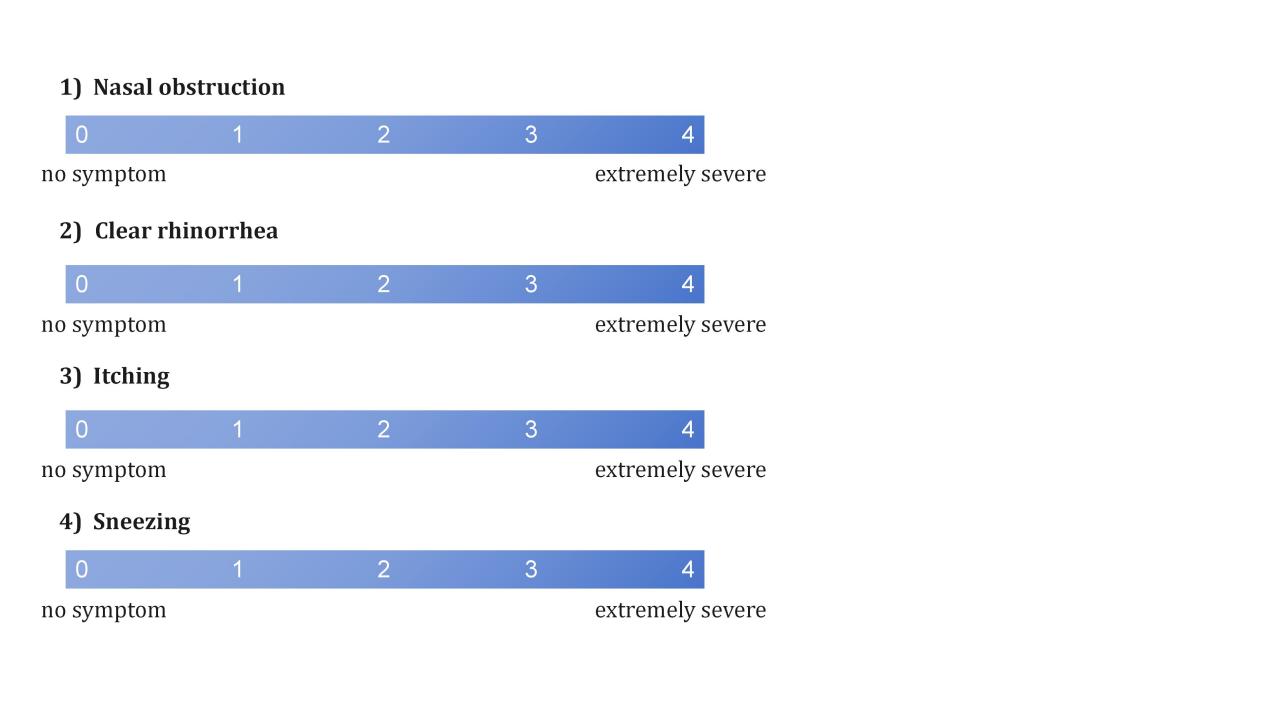
**

**Supplementary Figure 2.** TNSS score: 0–4 points (0 = no symptoms; 1 = mild; 2 = moderate; 3 = severe; 4 = extremely severe): mild: no symptoms causing obvious discomfort; moderate: symptoms causing discomfort but not affecting daily life or interfering with sleep; severe: symptoms interfering with daily life activities and sleep status, extremely severe: symptoms severely interfering with daily life activities and sleep status，adding up the points of each symptom to get the total score is the TNSS score.


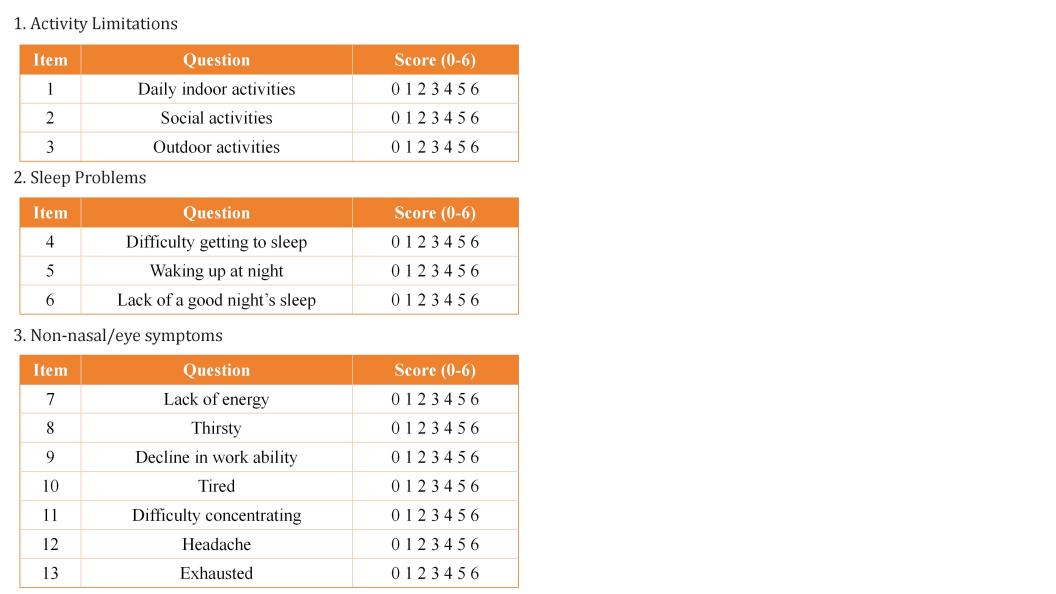


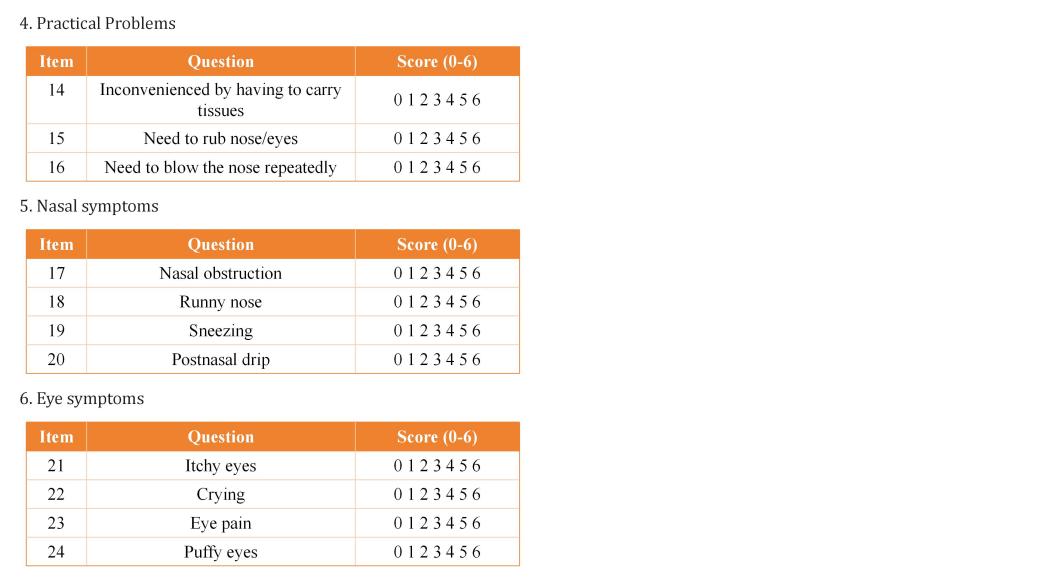


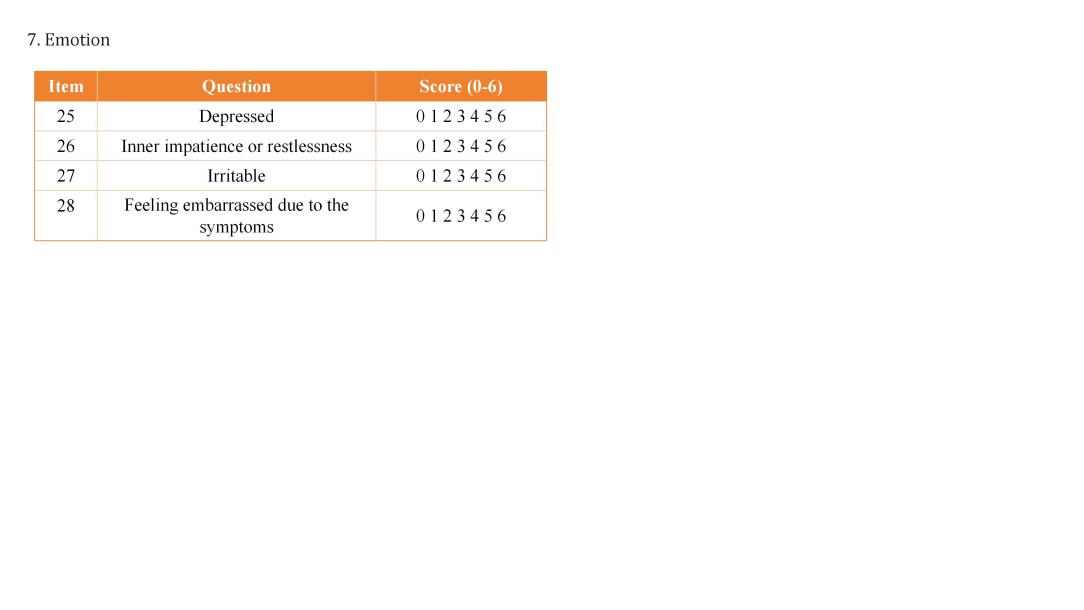


**Supplementary Figure 3.** RQLQ score: It consists of 7 dimensions and 28 items, each dimension is scored separately and the total score is the RQLQ score.

Scale: 0 = Not troubled; 1 = Hardly troubled at all; 2 = Somewhat troubled; 3 = Moderately troubled; 4 = Quite troubled; 5 = Very troubled; 6 = Extremely troubled

### Serum UHPLC-OE-MS untargeted metabolomics analysis

- **Metabolites extraction**

100 μL of sample was transferred to an EP tube. After the addition of 400 μL of extract

solution (methanol, containing isotopically-labelled internal standard mixture), the samples were vortexed for 30 s, sonicated for 10 min in ice-water bath, and incubated for 1 h at -40 ℃ to precipitate proteins. Then the sample was centrifuged at 12000 rpm(RCF=13800(×g),R= 8.6cm) for 15 min at 4 ℃. The resulting supernatant was transferred to a fresh glass vial for analysis. The quality control (QC) sample was prepared by mixing an equal aliquot of the supernatants from all of the samples.

- **LC-MS/MS analysis：**

LC-MS/MS analyses were performed using an ultra-high-performance liquid chromatography (UHPLC) system (Vanquish, Thermo Fisher Scientific) with a UPLC HSS T3 column (2.1 mm× 100 mm, 1.8 μm) coupled to Orbitrap Exploris 120 mass spectrometer (Orbitrap MS, Thermo). The mobile phase consisted of 5 mmol/L ammonium acetate and 5 mmol/L acetic acid in water (A) and acetonitrile (B). The auto-sampler temperature was 4 ℃, and the injection volume was 2 μL.

The Orbitrap Exploris 120 mass spectrometer was used for its ability to acquire MS/MS spectra on information-dependent acquisition mode in the control of the acquisition software (Xcalibur, Thermo). In this mode, the acquisition software continuously evaluates the full scan MS spectrum. The ESI source conditions were set as following: sheath gas flow rate as 50 Arb, Aux gas flow rate as 15 Arb, capillary temperature 320 ℃, full MS resolution as 60000, MS/MS resolution as 15000 collision energy as 10/30/60 in NCE mode, spray Voltage as 3.8 kV (positive) or -3.4 kV (negative), respectively.

- **Data preprocessing and annotation：**

The raw data were converted to the mzXML format using ProteoWizard and processed with an in-house program, which was developed using R and based on XCMS, for peak detection, extraction, alignment, and integration. Then an in-house MS2 database (BiotreeDB) was applied in metabolite annotation. The cutoff for annotation was set at 0.3.

### Bile acid targeted metabolomics analysis

Organic reagents including methanol (chromatography grade), acetonitrile (chromatography grade), and ultrapure water were procured from Thermo Fisher Scientific. Chemicals such as 2-pyrimidinylamine, triphenylphosphine, diphenylphosphinylazide, formic acid, and ammonium formate were obtained from Shanghai Aladdin.

- For sample preparation:

Transfer 50μL serum sample to a 1.5mL centrifuge tube, add 10μL bile acid internal standard mixture and 390μL methanol for extraction. Ultrasonicate for 10 minutes, then centrifuge at 12,000 r/min for 10 minutes at 10℃. Transfer 150μL supernatant to a new brown injection vial for testing.

- To prepare the quality control samples:

10 μL of supernatant from each biological sample was pipetted into a centrifuge tube and vortex-mixed at room temperature for 60 seconds.

- Bile acid chromatography Analysis

Bile acid metabolomic profiling was performed using a Vanquish ultra-high-performance liquid chromatography (UHPLC) system (Thermo Fisher Scientific, Waltham, MA, USA) equipped with a Phenomenex Kinetex C18 column (2.1×100 mm, 1.8μm). The mobile phases consisted of 0.1% formic acid + water + 10mM ammonium formatel and acetonitrile (phase B). Column temperature, 40℃; Sample chamber temperature ,10℃. Mass Spectrometry Parameters: The ACQUITY TQ-S triple quadrupole tandem mass spectrometer (Waters, USA) uses ESI as the ionization mode. Capillary voltage: 3.0 kV; Desolvent gas temperature: 500°C; Source temperature: 150°C; Desolvent gas flow rate: 900L/h; Cone-hole gas flow rate: 150L/h; Acquisition mode: Multiple Reaction Monitoring.

- Data collection

Data collection for this project utilizes MassLynx 4.1 software for both data acquisition and integration. Unless otherwise specified, the system automatically applies the integration method.

## Mouse model experiments

### Experimental animals and allergic rhinitis model.

**Supplementary Table 2.** Pathological scoring criteria

| Level 4 rating system | | |
| --- | --- | --- |
| Number of levels | - - 1. Type | - - 1. Instructions |
| 0 | Normal | Under normal research conditions, considering factors such as animal age, sex, and strain, the tissue is considered normal. Changes under other conditions may be considered abnormal. |
| 1 | Very Mild | Changes Slightly exceeding the normal range. |
| 2 | Mild | Lesions Lesions are observable but not severe. |
| 3 | Moderate | Lesions Obvious lesions, likely more severe. |
| 4 | Severe | Very severe (lesions have occupied the entire tissue or organ). |

[US] Peter Mann et al. International Norms for Pathological Changes and Diagnostic Criteria in Rats and Mice (INHAND).

Pathological scoring criteria.

# Results

## Clinical study

### Non-targeted metabolomic analysis revealed the metabolic profile and associated pathways of HDM-SCIT patients


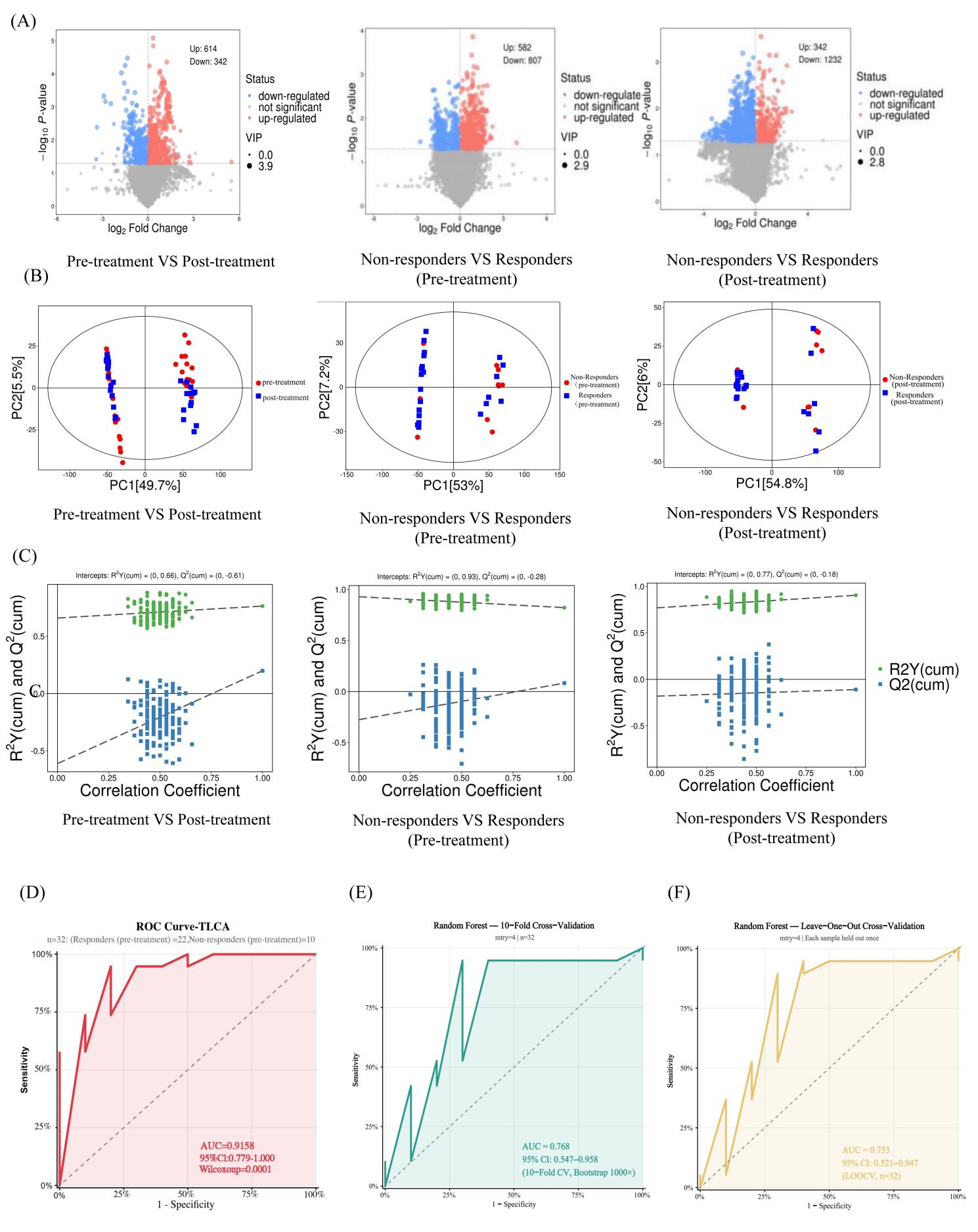


**Supplementary Figure 4.** Untargeted metabolomics profiles. (A)The volcano plot between different groups. (B) PCA score plot between different groups. (C) OPLS-DA permutation plot between different groups. (D) ROC curve of TLCA for predicting treatment response (responders: n=22, non-responders: n=10, total n=32; AUC=0.9158, 95% CI: 0.779–1.000, P=0.0001).(E) 10-fold cross-validation of the random forest model (mtry=4, n=32, 1000× bootstrap; AUC=0.768, 95% CI: 0.547–0.958).(F) Leave-one-out cross-validation of the random forest model (mtry=4, n=32; AUC=0.753, 95% CI: 0.521–0.947).

**Supplementary Table 3.** Details of 22 bile acids

| Name | Abbreviation | CAS | Article Number | Specifications |
| --- | --- | --- | --- | --- |
| Hyocholic Acid | HCA | 547-75-1 | S22143 | 5mg |
| Glycocholic acid | GCA | 475-31-0 | G131002 | 1g |
| Glycolithocholic acid | GLCA | 474-74-8 | MU-1018 | 50mg |
| Glycoursodeoxycholic acid | GUDCA | 64480-66-6 | MU-1016 | 50mg |
| [Glycochenodeoxycholic acid](https://www.chemsrc.com/en/cas/640-79-9_350673.html) | GCDCA | 16564-43-5 | S167765 | 1g |
| [Glycodeoxycholic acid](https://www.chemsrc.com/en/cas/360-65-6_248106.html) | GDCA | 360-65-6 | G113438 | 1g |
| Ursocholic acid | UCA | 2955-27-3 | U353408 | 10mg |
| Dioxolithocholic acid | DLCA | 517-33-9 | H336519 | 5mg |
| Ursodeoxycholic acid | UDCA | 128-13-2 | U110695 | 5g |
| Chenodeoxycholic acid | CDCA | 474-25-9 | C104903 | 20mg |
| Sodium deoxycholate | DCA | 302-95-4 | D6128 | 10g |
| Lithocholic acid | LCA | 434-13-9 | L106779 | 1g |
| Allocholic acid | ACA | 2464-18-8 | A336073 | 5mg |
| 12-Ketochenodeoxycholic acid | 12-KCDCA | 2458-08-4 | HY-W403933 | 1 mg |
| Murideoxycholic acid | MDCA | 668-49-5 | C336515 | 2mg |
| Cholic acid | CA | 81-25-4 | C103692 | 20mg |
| Taurocholic acid | TCA | 81-24-3 | T336942 | 1g |
| Taurochenodeoxycholic acid sodium salt | TCDCA | 6009-98-9 | MU-1024 | 50mg |
| Taurine hyodeoxycholic acid | THDCA | 2958-04-5 | 111943-202303 | 20mg |
| Sodium taurodeoxycholate hydrate | TDCA | 207737-97-1 | S168485 | 1g |
| Tauroursodeoxycholic acid | TUDCA | 14605-22-2 | T614340 | 100 μg |
| Taurolithocholic acid sodium salt | TLCA | 6042-32-6 | MU-1028 | 25mg |

**
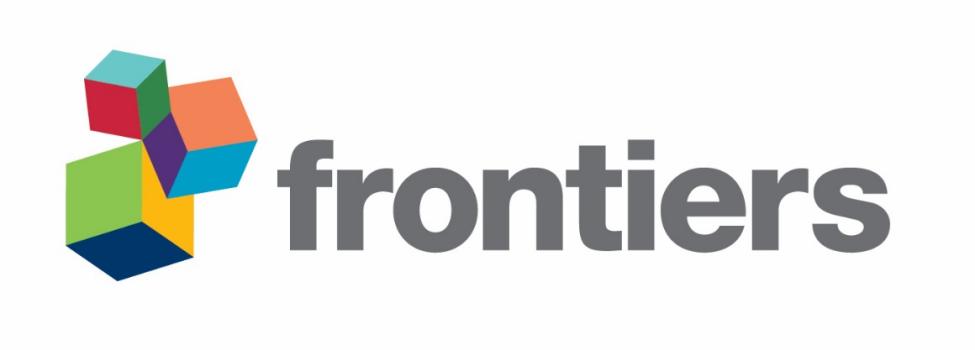
**
