# Supplementary material for: Serum metabolomic profiling identifies taurolithocholic acid as a predictor of HDM-SCIT response in allergic rhinitis: clinical discovery and experimental validation
Source: Front Immunol. 2026 May 11;17:1822573. doi: 10.3389/fimmu.2026.1822573 (PMC13199366; doi:10.3389/fimmu.2026.1822573)
Supplement: Supplementary file 3 [file Presentation2.pptx]

## Slide 1
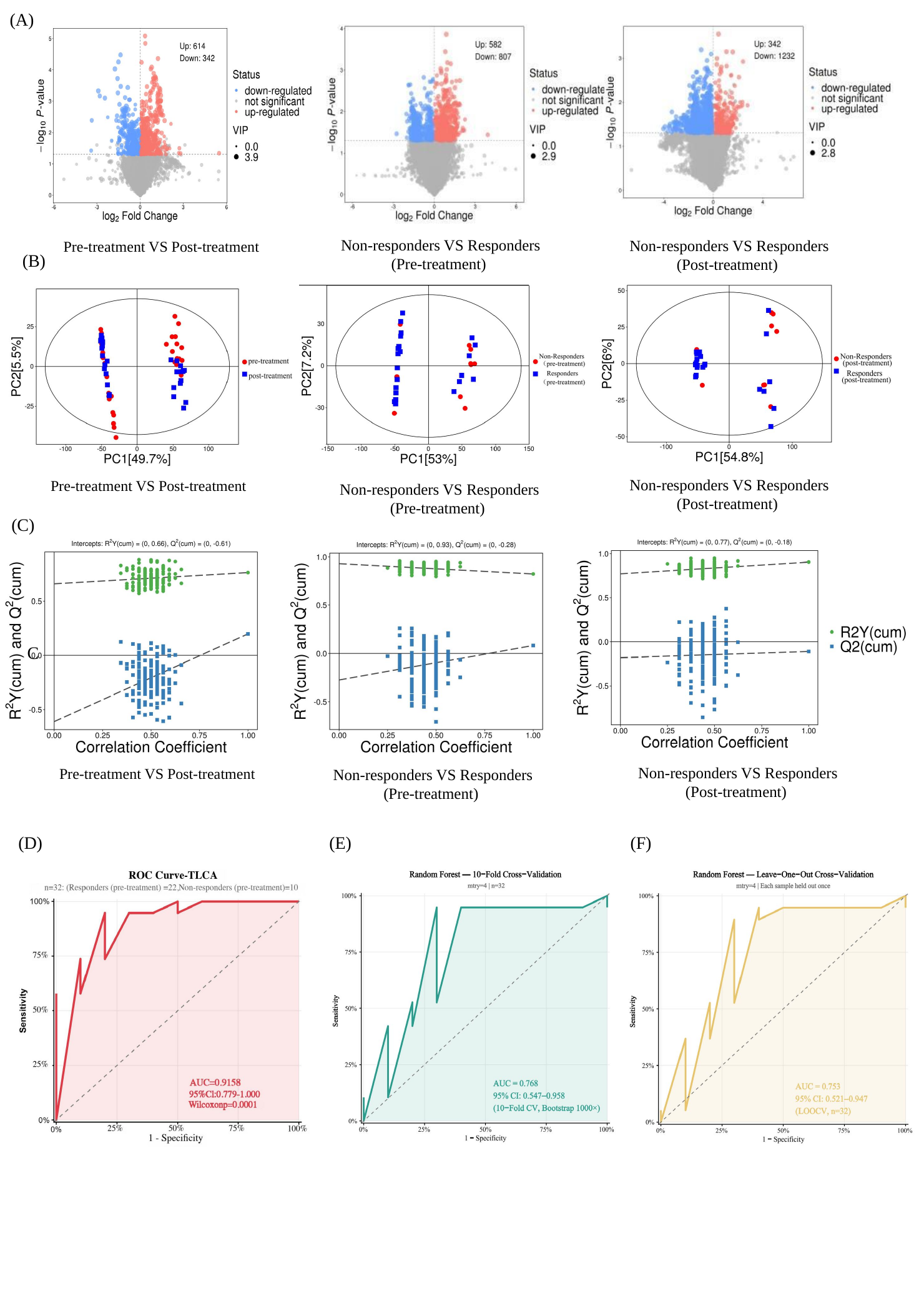

(A)
Non-responders VS Responders
(Pre-treatment)
Non-responders VS Responders
(Post-treatment)
 Pre-treatment VS Post-treatment
(B)
Non-responders VS Responders
(Post-treatment)
 Pre-treatment VS Post-treatment
Non-responders VS Responders
(Pre-treatment)
(C)
C
Non-responders VS Responders
(Post-treatment)
 Pre-treatment VS Post-treatment
Non-responders VS Responders
(Pre-treatment)
(D)
(E)
(F)
